# Stabilization of zwitterionic versus canonical proline by water molecules

**DOI:** 10.1186/s40064-015-1661-8

**Published:** 2016-01-06

**Authors:** Gang Yang, Lijun Zhou, Yang Chen

**Affiliations:** College of Resource and Environment and Chongqing Key Laboratory of Soil Multi-scale Interfacial Process, Southwest University, 400715 Chongqing, People’s Republic of China; College of Chemistry Chemical Engineering and Environmental Engineering, Liaoning Shihua University, 113001 Fushun, Liaoning People’s Republic of China

**Keywords:** Density functional calculations, Conformational analysis, Zwitterionic stabilization, H-bonds

## Abstract

At physiological conditions, a majority of biomolecules (e.g., amino acids, peptides and proteins) exist predominantly in the zwitterionic form that usually decides the biological functions. However, zwitterionic amino acids are not geometrically stable in gas phase and this seriously hampers the understanding of their structures, properties and biological functions. To this end, one of the recent research focuses is to demonstrate the stabilization effects of zwitterionic amino acids. Relative stabilities of canonical conformers are dependent on water contents, while zwitterionic stability improves monotonously and pronouncedly with increase of water contents. We find that one water molecule can render zwitterionic proline geometrically stable, and stabilities of different zwitterionic amino acids increase as glycine <proline <arginine. In addition, we have determined the numbers of water molecules required for zwitterionic proline to be energetically preferential and conformationally predominant, respectively as four and five. Five water molecules are enough to fill up the first shell of proline functional sites (carboxylic and amido), which is in line with the results of glycine. At any water content, zwitterionic formation will not be hindered kinetically because of rather low activation barriers, and the distribution of zwitterionic amino acids will be largely dependent on their thermodynamic stabilities.

**Electronic supplementary material:**

The online version of this article (doi:10.1186/s40064-015-1661-8) contains supplementary material, which is available to authorized users.

## Background

In aqueous solutions, a wide range of biomolecules (e.g., amino acids, peptides and proteins) exist predominantly in the zwitterionic form, and water molecules are essential to maintain their native conformations and physiological functions (Timasheff 1970; Rand 2004). Interaction of biomolecules and water has attracted general interest (Teeter 1991; Levy and Onuchic 2004; Corradini et al. 2013), and amino acids, the structural unit of proteins, are generally the protypes for ab initio and density functional investigation of peptides and proteins (Császár 1992; Hu et al. 1995; Yu et al. 1995; Zhang and Chung-Phillips 1998; Remko and Rode 2001; Hoyau and Ohanessian 1998; Ai et al. 2003; Constantino et al. 2005; Remko and Rode 2006).

On the other hand, amino acids in gas phase consist entirely of canonical conformers (Császár 1992; Hu et al. 1995; Yu et al. 1995), which is totally different from the condition in aqueous solutions. This is obviously an obstacle for us to comprehend the electronic properties and biological functions of zwitterionic structures. Zwitterions can generate strong electric fields around that usually decide the biological functions of biomolecules. Owing to the particular importance, one of the recent research focuses is to demonstrate the stabilization effects of zwitterionic amino acids (Yu et al. 1995; Zhang and Chung-Phillips 1998; Remko and Rode 2001; Hoyau and Ohanessian 1998; Ai et al. 2003; Constantino et al. 2005; Remko and Rode 2006; Corral et al. 2006; Yang et al. 2009; Jensen and Gordon 1995; Snoek et al. 2002; Yamabe et al. 2003; Balta and Aviyente 2004; Im et al. 2008; Gutowski et al. 2000; Rimola et al. 2008; Wu and McMahon 2007; Rimola et al. 2013; Kass 2005; Yang et al. 2008; Tian et al. 2009; Hwang et al. 2011; Li et al. 2011; Kim et al. 2014; Yang and Zhou 2014), and a variety of attempts have been made in this aspect, such as protonation and deprotonation (Yu et al. 1995; Zhang and Chung-Phillips 1998), metalation (Hoyau and Ohanessian 1998; Ai et al. 2003; Constantino et al. 2005; Remko and Rode 2006; Corral et al. 2006; Yang et al. 2009), hydration (Jensen and Gordon 1995; Snoek et al. 2002; Yamabe et al. 2003; Balta and Aviyente 2004; Im et al. 2008; Hwang et al. 2011; Li et al. 2011; Kim et al. 2014) and anion attachment (Kass 2005; Yang et al. 2008; Tian et al. 2009). A minimum of two water molecules is required to stabilize glycine in the zwitterionic form (Jensen and Gordon 1995), while one is enough to cause zwitterionic arginine as the lowest-energy conformer (Im et al. 2008). Interaction of proline and water has been investigated by different groups (Li et al. 2011; Kim et al. 2014), and two water molecules were considered necessary for rendering zwitterionic proline to be geometrically stable.

In this work, density functional calculations were employed to comprehend the gas-phase interaction of different proline conformers and water with a wide range of contents (n = 0–5). Five water molecules were found enough to fill up the first shell of proline functional sites (carboxylic and amido), as in the case of glycine (Kokpol et al. 1988). We determined that one water molecule can stabilize proline in zwitterionic form; meanwhile, stabilities of zwitterionic glycine, proline and arginine were compared with each other. Then, the numbers of water molecules necessary to render zwitterionic proline energetically preferential and conformationally predominant were respectively determined, and the relationship of zwitterionic stability vs. water content was demonstrated. Finally, transition states for the transformation from canonical to zwitterionic proline at all water contents were located, testifying that zwitterionic formation in gas phase is impeded mainly by the thermodynamic rather than kinetic stability.

## Computational methods

B3LYP density functional, in combination with 6-31+G (d,p) basis set, was used for structural optimizations and frequency calculations (Frisch et al. 2013). Energy minima were confirmed to have all positive frequencies while transition states displayed a single imaginary frequency corresponding to the eigenvector along the reaction path, and the assignment of each transition sate was verified by perturbing the structure along both directions of products and reactants with subsequent structural optimizations. Single-point energy calculations at the B3LYP/6-311++G (2df, 2pd) and MP2/6-311++G (2df, 2pd) levels of theory were then carried out on these optimized structures. Afterwards, the 6-31+G(d,p) and 6-311++G (2df, 2pd) basis sets were respectively designated as bs1 and bs2, and unless otherwise noted, all energies were reported at the B3LYP/bs2//B3LYP/bs1 level, which has been sufficiently validated before (Li et al. 2011; Kim et al. 2014; Yang et al. 2009; Rankin et al. 2002; Yang et al. 2010; Ajitha and Suresh 2011) and in this work.

## Results and discussion

**Scheme 1:**
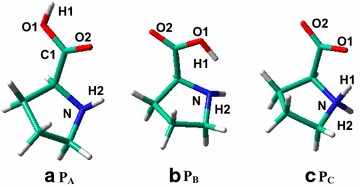
Proline in canonical (**a** and **b**) and zwitterionic (**c**) forms

As shown in Scheme 1, three proline conformers have been considered in this work: two canonical (**P**_**A**_ and **P**_**B**_) while the third zwitterionic (**P**_**C**_), which are in line with previous studies (Rankin et al. 2002; Yang et al. 2010; Ajitha and Suresh 2011; Yang et al. 2015). **P**_**A**_ and **P**_**B**_ are canonical conformers that predominant in gas phase (Eszter et al. 2003; Alln et al. 2004; Kapitán et al. 2006) and become the choice for catalytic studies (Rankin et al. 2002; Yang et al. 2010; Ajitha and Suresh 2011). **P**_**A**_ is slightly less stable than **P**_**B**_ with the relative energy of 1.6 kcal/mol (Table 1 and Additional file 1: Table S1). **P**_**C**_ (zwitterionic) does not represent a local minimum on the potential energy surface (PES) (Eszter et al. 2003; Alln et al. 2004), and so its relative stability is evaluated by fixing the N-H_1_ distance at 1.030 Å, with production of a higher relative energy than **P**_**B**_ (13.1 kcal/mol). Relative energies of these proline conformers are also calculated at other theoretical levels, which are in line with the default B3LYP/bs2//B3LYP/bs1 method (Additional file 1: Table S1).

**Table 1:** Relative stabilities for interacted structures of different proline conformers (*E*_RS_) and water with a wide range of contents (n = 0–5)

		n = 0^a^	n = 1	n = 2	n = 3	n = 4	n = 5
Gas phase	**P**_**A**_ **nW**_**I**_	1.6	−1.2	−3.3	−0.1	0.4	−0.5
	**P**_**C**_ **nW**_**I**_	13.1	8.7	2.8	1.1	−2.6	−6.0

Energy units in kcal/mol

For a given water content, **P**_**B**_ **nW**_**I**_ is used as energy benchmark

^a^In zwitterionic proline (**P**_**C**_), the N-H_1_ distance is fixed at 1.030 Å during structural optimizations

For each proline confirmer, interactions with water can result in the various structures that are discerned by suffixing **nW**_**N**_, wherein n (n = 1, 2, …) represents the number of water molecules and N (=I, II, …) ranks the stability of different structures. For instance, **P**_**A**_**2W**_**III**_/**P**_**C**_**5W**_**I**_ stands for the third/first stable conformer for **P**_**A**_/**P**_**C**_ interactions with two/five water molecules.

### Necessary for zwitterionic stabilization

**Fig. 1:**
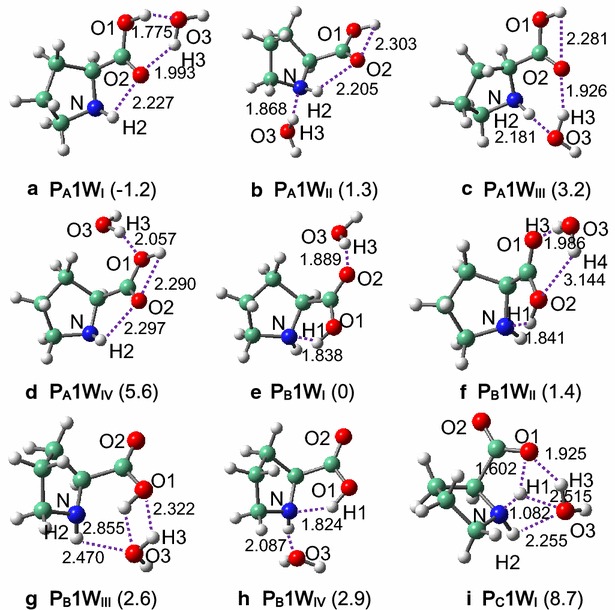
Interacted structures of proline conformers (**P**_**A**_, **P**_**B**_ and **P**_**C**_) with one water molecule. Relative energies (kcal/mol) are given in parentheses, using **P**_**B**_ **1W**_**I**_ as benchmark. H-bonds (Å) are marked with *dashed lines*

Figure 1 shows the interacted structures of proline conformers (**P**_**A**_, **P**_**B**_ and **P**_**C**_) and one water molecule. **P**_**A**_ and **P**_**B**_ each result in four stable interacted structures, and their relative stabilities increase in the order of **P**_**A**_**1W**_**IV**_ (5.6) <**P**_**A**_**1W**_**III**_ (3.2) <**P**_**B**_**1W**_**IV**_ (2.9) <**P**_**B**_**1W**_**III**_ (2.6) <**P**_**B**_**1W**_**II**_ (1.4) <**P**_**A**_**1W**_**II**_ (1.3) <**P**_**B**_**1W**_**I**_ (0) <**P**_**A**_**1W**_**I**_ (−1.2), see Table 1 and Additional file 1: Table S2. Note that relative energies (unit kcal/mol) are given in parentheses, and **P**_**B**_**1W**_**I**_ is used as benchmark. It can be seen that the conformational preference is altered by presence of one water molecule, and the lowest-energy structure is from **P**_**A**_ instead of **P**_**B**_ that represents the most stable conformer in the isolated state (Eszter et al. 2003; Alln et al. 2004). In **P**_**A**_**1W**_**I**_, water forms two H-bonds with the carboxylic site of proline (O_1_H_1_•••O_3_ and O_3_H_3_•••O_2_). Both **P**_**A**_**1W**_**II**_ and **P**_**A**_**1W**_**IV**_ have only one intermolecular H-bond, while H-bonding interactions of the former is apparently stronger as reflected from their distances (O_3_H_3_•••N: 1.868 Å vs. O_3_H_3_•••O_1_: 2.057 Å). In **P**_**B**_**1W**_**I**_, water interacts mainly with one of the two carboxylic-O atoms, while structures involving both carboxylic-O atoms cannot be located as energy minima. The less efficient intermolecular H-bonding interactions in **P**_**B**_**1W**_**I**_ cause it to have a higher relative energy than **P**_**A**_**1W**_**I**_.

It is interesting to find that one water molecule can stabilize proline in the zwitterionic form. In **P**_**C**_**1W**_**I**_, two H-bonds are constructed for water with the carboxylic and amido sites of proline (NH_2_•••O_3_ and O_3_H_3_•••O_1_) that resemble the condition in **P**_**B**_**1W**_**III**_; however, both H-bonds have been significantly reinforced, with the distances being optimized respectively at 2.255 and 1.925 Å vs. 2.470 and 2.322 Å in **P**_**B**_**1W**_**III**_. Despite that, **P**_**C**_**1W**_**I**_ still has a much higher energy than **P**_**B**_**1W**_**I**_ (8.7 kcal/mol, see Table 1 and Additional file 1: Table S2). For glycine, arginine and proline, relative stabilities of their zwitterionic conformers should differ from each other. Both zwitterionic proline and arigine require one water molecule in order to remain geometrically stable, while zwitterionic glycine necessitates two water molecules (Jensen and Gordon 1995; Im et al. 2008). In addition, one water molecule is already sufficient to cause zwitterionic arginine as the global energy minimum (Im et al. 2008). Accordingly, relative stabilities of these zwitterionic conformers should increase in the order as glycine <proline <arginine.

### Introduction of more water molecules

**Fig. 2:**
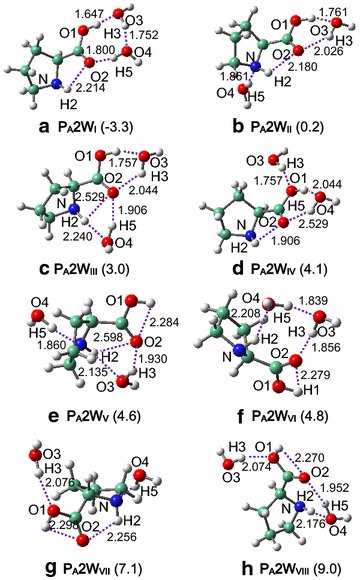
Interacted structures of **P**_**A**_ and two water molecules. Relative energies (kcal/mol) are given in parentheses, using **P**_**B**_ **2W**_**I**_ as benchmark. H-bonds (Å) are marked with *dashed lines*

**Fig. 3:**
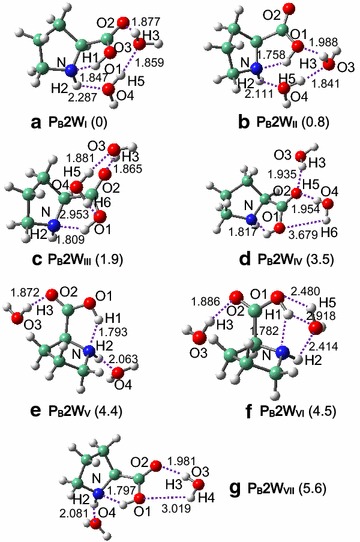
Interacted structures of **P**_**B**_ and two water molecules. Relative energies (kcal/mol) are given in parentheses, using **P**_**B**_ **2W**_**I**_ as benchmark. H-bonds (Å) are marked with *dashed lines*

**Fig. 4:**
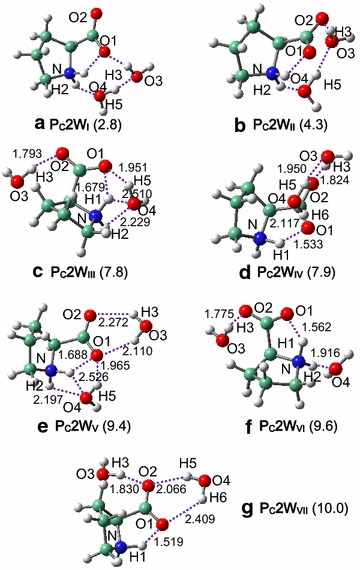
Interacted structures of **P**_**C**_ and two water molecules. Relative energies (kcal/mol) are given in parentheses, using **P**_**B**_ **2W**_**I**_ as benchmark. H-bonds (Å) are marked with *dashed lines*

Introduction of a second water molecule to proline results in a more conformational diversity, and **P**_**A**_, **P**_**B**_ and **P**_**C**_ respectively result in 8, 7 and 7 interacted structures, see Figs. 2, 3, 4 with their relative energies being listed in Table 1 and Additional file 1: Table S3. Structure (i.e., **P**_**A**_**2W**_**I**_) corresponding to **P**_**A**_ remains the most stable as in the case of n = 1, wherein two water molecules are both situated at the carboxylic site of proline. Although with two resembling intermolecular H-bonds, interactions in **P**_**A**_**2W**_**I**_ are pronouncedly stronger than in **P**_**A**_**1W**_**I**_ (1.647 and 1.800 Å vs. 1.775 and 1.993 Å). Structures of two water molecules may be combinatorially obtained from those of one water molecule; e.g., combination of **P**_**A**_**1W**_**I**_ and **P**_**A**_**1W**_**II**_ leads to **P**_**A**_**2W**_**II**_.

In **P**_**B**_**2W**_**I**_ and **P**_**B**_**2W**_**II**_, one water molecule forms H-bond with the carboxylic-O site of proline while the other forms H-bond with the amido site, and two water molecules are connected with each other by one strong H-bond. In **P**_**C**_**2W**_**I**_, the intermolecular H-bonding interactions between proline and water resemble those in **P**_**B**_**2W**_**II**_ and also in **P**_**C**_**1W**_**I**_ while are greatly consolidated as compared to **P**_**C**_**1W**_**I**_, and the zwitterionic stability improves accordingly (Table 1). Unlike the case of one water, zwitterions (**P**_**C**_**2W**_**IV**_ and **P**_**C**_**2W**_**VII**_) that have H-bonds only at the carboxylic site of proline remain geometrically stable, as a result of enhanced intermolecular interactions.

**Scheme 2:**
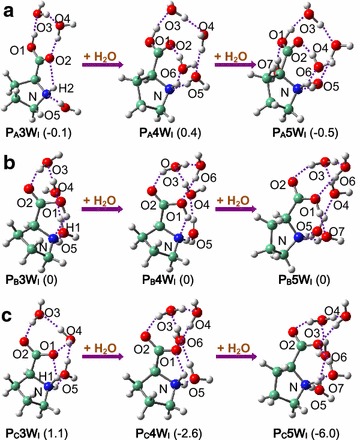
Gradual increase of water to interact with different proline conformers: **a** **P**_**A**_ **3W**_**I**_ → **P**_**A**_ **4W**_**I**_ → **P**_**A**_ **5W**_**I**_, **b** **P**_**B**_ **3W**_**I**_ → **P**_**B**_ **4W**_**I**_ → **P**_**B**_ **5W**_**I**_ and **c** **P**_**C**_ **3W**_**I**_ → **P**_**C**_ **4W**_**I**_ → **P**_**C**_ **5W**_**I**_. Relative energies (kcal/mol) are given in parentheses, using **P**_**B**_ **nW**_**I**_ as benchmark (n = 3, 4, 5). H-bonds are marked with *dashed lines*

Scheme 2 and Additional file 1: Figures S1–S8 show that with increase of water contents, the functional sites (carboxylic and amido) of proline are gradually saturated. Some structures of **P**_**B**_ and **P**_**C**_ interactions with 2–4 water molecules have been reported by Lee et al. (Kim et al. 2014) that are in good agreement with the present results. It indicates that five water molecules are enough to fill up the first shell of proline functional sites (carboxylic and amido), consistent with the results of glycine (Kokpol et al. 1988). Relative energies in Table 1 and Additional file 1: Table S3–S6 demonstrate that the leading position of **P**_**A**_ begins to be challenged since the third water molecule. **P**_**B**_**3W**_**I**_ has comparable stability with **P**_**A**_**3W**_**I**_, while **P**_**B**_**4W**_**I**_ rather than **P**_**A**_**4W**_**I**_ is slightly energetically preferential. Instead, stability of zwitterionic proline improves monotonously and pronouncedly with the gradual increase of water contents. Zwitterionic structures ascend to be the most stable configuration at n = 4 (**P**_**C**_**4W**_**I**_) and becomes the predominant configuration since n = 5 (**P**_**C**_**5W**_**I**_), where rather complex H-bonding networks are constructed between water and proline.

### Transformation to zwitterionic proline

Transition state structures for the transformation from canonical to zwitterionic proline with presence of different water contents (n = 1–5) are shown in Additional file 1: Figures S9–S13, and their activation barriers (*E*_A_) and reaction heats (*E*_R_) are listed in Table 2. In transition state structures, O-H_1_ bonds have already ruptured while N-H_1_ bonds are being constructed. At a given water content, there may exist several zwitterionic structures, and the activation barriers (*E*_A_) that are dependent on reaction paths may differ significantly; e.g., at n = 3, the *E*_A_ values vary in the range of 2.6–8.6 kcal/mol, and the maximum (8.6 kcal/mol) is even larger than those of n = 1 and 2. Generally, with increase of water contents, transformation to zwitterionic structures becomes more thermodynamically favourable, as indicated by the calculated reaction heats (*E*_R_), see Table 2.

**Table 2:** Activation barriers (*E*_A_) and reaction heats (*E*_R_) for transformation from canonical (**P**_**B**_) to zwitterionic (**P**_**C**_) proline with presence of water molecules

Reaction paths	*E*_A_	*E*_R_
One water molecule		
**P**_**B**_ **1W**_**III**_ → [**TS1W**_**III–I**_]^≠^ → **P**_**C**_ **1W**_**I**_	6.7	6.1
Two water molecules		
**P**_**B**_ **2W**_**II**_ → [**TS2W**_**II–I**_]^≠^ → **P**_**C**_ **2W**_**I**_	4.2	2.0
**P**_**B**_ **2W**_**I**_ → [**TS2W**_**I–II**_]^≠^ → **P**_**C**_ **2W**_**II**_	6.1	4.3
**P**_**B**_ **2W**_**VI**_ → [**TS2W**_**VI–III**_]^≠^ → **P**_**C**_ **2W**_**III**_	4.9	3.2
**P**_**B**_ **2W**_**III**_ → [**TS2W**_**III–IV**_]^≠^ → **P**_**C**_ **2W**_**IV**_	6.4	6.0
**P**_**B**_ **2W**_**VII**_ → [**TS2W**_**VII–V**_]^≠^ → **P**_**C**_ **2W**_**V**_	6.2	3.8
**P**_**B**_ **2W**_**V**_ → [**TS2W**_**V–VI**_] → **P**_**C**_ **2W**_**VI**_	5.8	5.2
**P**_**B**_ **2W**_**IV**_ → [**TS2W**_**IV–VII**_]^≠^ → **P**_**C**_ **2W**_**VII**_	6.7	6.5
Three water molecules		
**P**_**B**_ **3W**_**II**_ → [**TS3W**_**II–I**_]^≠^ → **P**_**C**_ **3W**_**I**_	3.8	0.7
**P**_**B**_ **3W**_**I**_ → **[TS3W**_**I–II**_ **]** ^≠^ → **P**_**C**_ **3W**_**II**_	4.3	1.9
**P**_**B**_ **3W**_**III**_ → **[TS3W**_**III–III**_ **]** ^≠^ → **P**_**C**_ **3W**_**III**_	6.3	−1.1
**P**_**B**_ **3W**_**III**_ → **[TS3W**_**III–IV**_ **]** ^≠^ → **P**_**C**_ **3W**_**IV**_	8.6	0.7
**P**_**B**_ **3W**_**IV**_ → **[TS3W**_**IV–V**_ **]** ^≠^ → **P**_**C**_ **3W**_**V**_	2.6	−1.1
Four water molecules		
**P**_**B**_ **4W**_**I**_ → **[TS4W**_**I–I**_ **]** ^≠^ → **P**_**C**_ **4W**_**I**_	1.9	−2.6
**P**_**B**_ **4W**_**II**_ → **[TS4W**_**II–I**_ **]** ^≠^ → **P**_**C**_ **4W**_**I**_	5.8	−3.4
**P**_**B**_ **4W**_**II**_ → **[TS4W**_**II–III**_ **]** ^≠^ → **P**_**C**_ **4W**_**III**_	5.3	−2.4
**P**_**B**_ **4W**_**III**_ → **[TS4W**_**III–IV**_ **]** ^≠^ → **P**_**C**_ **4W**_**IV**_	2.7	−2.1
Five water molecules		
**P**_**B**_ **5W**_**I**_ → **[TS5W**_**I–I**_ **]** ^≠^ → **P**_**C**_ **5W**_**I**_	4.2	−6.0
**P**_**B**_ **5W**_**II**_ → **[TS5W**_**II–II**_ **]** ^≠^ → **P**_**C**_ **5W**_**II**_	4.8	−6.4

Energy units in kcal/mol

**Fig. 5:**
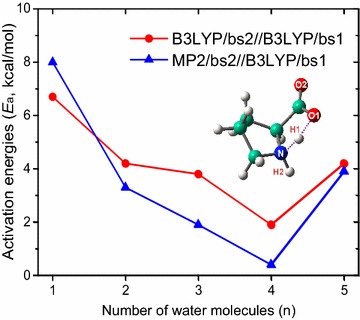
Activation energies (*E*_A_) for the transformation to zwitterions (**P**_**C**_ **nW**_**I**_) in presence of water molecules (n = 1–5) calculated at different levels of theory. Two reaction paths can lead to **P**_**C**_ **4W**_**I**_ (Table 2), and the path with the lower activation barrier is given here

Figure 5 gives the activation barriers (*E*_A_) for transformation to the most stable zwitterionic structures (**P**_**C**_**nW**_**I**_) (n = 1–5), which are equal to 6.7, 4.2, 3.8, 1.9 and 4.2 kcal/mol for n = 1, 2, 3, 4 and 5, respectively (Table 2). The larger *E*_A_ value for **P**_**C**_**5W**_**I**_ than for **P**_**C**_**4W**_**I**_ is due to the alteration of direct transformation path from O_1_ to N and the involvement of one water molecule (H_11_O_7_H_12_) (Yamabe et al. 2003). The changing trends of *E*_A_ at MP2/bs2//B3LYP/bs1 level are identical to that of B3LYP/bs2//B3LYP/bs1; in addition, the exact *E*_A_ values of these two methods are rather close each other, thus validating the default methodologies. At any water content, the activation barriers (*E*_A_) for transformation from canonical to zwitterionic structures are rather low, and accordingly the transformation to zwitterionic structures in gas phase will not be kinetically hindered. It is thus assumed that the zwitterionic distribution at a given water content (n ≥ 0) are determined principally by their thermodynamic stabilities.

## Conclusions

Conformational analyses are performed for interacted structures of proline conformers (**P**_**A**_, **P**_**B**_ and **P**_**C**_) and water with a wide range of contents (n = 1–5). Five water molecules are enough to fill up the first shell of proline functional sites (carboxylic and amido). The conformational preferences of proline are altered by gradual increase of water contents, and intermolecular H-bonds play a crucial role in deciding their relative stabilities.

It is interesting to find that one water molecule has already rendered zwitterionic proline to be geometrically stable with formation of two strong intermolecular H-bonds. Unlike the canonical conformers, the relative stabilities of zwitterionic proline improve monotonously and pronouncedly with gradual increase of water contents. Zwitterionic proline is energetically preferential over canonical structures at n = 4 and conformationally predominant at n = 5; in these cases, rather complex H-bonding networks are constructed between water and proline. Zwitterionic stabilities of different amino acids differ substantially and increase in the order of glycine <proline <arginine.

At a given water content, the activation barriers for transformation from canonical to zwitterionic conformers are significantly dependent on reaction paths; nonetheless, the activation barriers are rather low for all water contents. It is thus demonstrated that the zwitterionic distribution, at any water content (n ≥ 0), will be determined mainly by the thermodynamic rather than kinetic factor.

## Additional file

10.1186/s40064-015-1661-8 Relative stabilities for different proline conformers with presence of 0, 1, 2, 3, 4 and 5 water molecules (**Tables S1**–**S5**) as well as structures of proline conformers with presence of 3, 4 and 5 water molecules (**Figures S1**–**S8**) and transition states for the conformational transformation from canonical to zwitterionic proline with presence of 1, 2, 3, 4, 5 water molecules (**Figures S9**–**S13**).
