# Supplementary material for: Stabilization of zwitterionic versus canonical proline by water molecules
Source: Springerplus. 2016 Jan 6;5:19. doi: 10.1186/s40064-015-1661-8 (PMC4703596; doi:10.1186/s40064-015-1661-8)
Supplement: Supplementary file 1 — 10.1186/s40064-015-1661-8 Relative stabilities for different proline conformers with presence of 0, 1, 2, 3, 4 and 5 water molecules (Tables S1–S5) as well as structures of proline conformers with presence of 3, 4 and 5 water molecules (Figures S1–S8) and transition states for the conformational transformation from canonical to zwitterionic proline with presence of 1, 2, 3, 4, 5 water molecules (Figures S9–S13). [file 40064_2015_1661_MOESM1_ESM.doc]

# Supplementary Information For

**Stabilization of Zwitterionic versus Canonical Proline by Water Molecules**

Gang Yang,^1,*^ Lijun Zhou,^1^ and Yang Chen^2^

^1^ College of Resource and Environment & Chongqing Key Laboratory of Soil Multi-scale Interfacial Process, Southwest University, 400715, Chongqing, P.R. China

^2^ College of Chemistry Chemical Engineering and Environmental Engineering, Liaoning Shihua University, 113001, Liaoning, P.R. China

| **Table S1.** Relative energies for different proline conformers obtained at the various theoretical levels*^a^*,*^b^* | | | | |
| --- | --- | --- | --- | --- |
|  | B3LYP/bs2//B3LYP/bs1 | B3LYP/bs2 | MP2/bs2//B3LYP/bs1 | MP2/bs2//MP2/bs1 |
| **P_A_** | 1.6 | 1.6 | 2.1 | 2.0 |
| **P_B_** | 0 | 0 | 0 | 0 |
| **P_C_*^d^*** | 13.1 | 13.1 | 13.2 | 13.1 |
| *^a^* Energy units in kcal/mol;  *^b^* **P_B_** as the energy benchmark for each case;  *^c^* In zwitterionic proline (**P_C_**), the N-H_1_ distance is fixed at 1.030 Å during structural optimizations. | | | | |

| **Table S2.** Relative energies for interacted structures of proline conformers with one water molecule*^a^*,*^b^* | | | | | | | | | |
| --- | --- | --- | --- | --- | --- | --- | --- | --- | --- |
|  | **P_A_1W_I_** | **P_A_1W_II_** | **P_A_1W_III_** | **P_A_1W_IV_** | **P_B_1W_I_** | **P_B_1W_II_** | **P_B_1W_III_** | **P_B_1W_IV_** | **P_C_1W_I_** |
| Gas phase | -1.2 | 1.3 | 3.2 | 5.6 | 0 | 1.4 | 2.6 | 2.9 | 8.7 |
| PCM model | 2.0 | 2.0 | 5.2 | 6.5 | 0 | -0.6 | 3.0 | 1.3 | 0.6 |
| *^a^* Energy units in kcal/mol;  *^b^* **P_B_1W_I_** as the energy benchmark for each case. | | | | | | | | | |

| **Table S3.** Relative energies for interacted structures of proline conformers with two water molecules (X = A, B, C)*^a^*,*^b^* | | | | | | | | |
| --- | --- | --- | --- | --- | --- | --- | --- | --- |
|  | **2W_I_** | **2W_II_** | **2W_III_** | **2W_IV_** | **2W_V_** | **2W_VI_** | **2W_VII_** | **2W_VIII_** |
| **P_A_** | -3.3 | 0.2 | 3.0 | 4.1 | 4.6 | 4.8 | 7.1 | 9.0 |
| **P_B_** | 0 | 0.8 | 1.9 | 3.5 | 4.4 | 4.5 | 5.6 |  |
| **P_C_** | 2.8 | 4.3 | 7.8 | 7.9 | 9.4 | 9.6 | 10.0 |  |
| *^a^* Energy units in kcal/mol;  *^b^* **P_B_2W_I_** as the energy benchmark. | | | | | | | | |

| **Table S4.** Relative energies for interacted structures of proline conformers with three water molecules*^a^*,*^b^* | | | | | | |
| --- | --- | --- | --- | --- | --- | --- |
|  | **3W_I_** | **3W_II_** | **3W_III_** | **3W_IV_** | **3W_V_** | **3W_VI_** |
| **P_A_** | -0.1 | 2.8 | 3.7 | 5.8 | 7.6 | 9.8 |
| **P_B_** | 0 | 0.4 | 3.2 | 5.2 |  |  |
| **P_C_** | 1.1 | 1.9 | 2.1 | 3.9 | 4.1 |  |
| *^a^* Energy units in kcal/mol;  *^b^* **P_B_3W_I_** as the energy benchmark. | | | | | | |

| **Table S5.** Relative energies for interacted structures of proline conformers with four water molecules*^a^*,*^b^* | | | | |
| --- | --- | --- | --- | --- |
|  | **4W_I_** | **4W_II_** | **4W_III_** | **4W_IV_** |
| **P_A_** | 0.4 | 4.3 | 7.2 |  |
| **P_B_** | 0 | 2.1 | 2.4 |  |
| **P_C_** | -2.6 | -1.3 | -0.3 | 0.3 |
| *^a^* Energy units in kcal/mol;  *^b^* **P_B_4W_I_** as the energy benchmark. | | | | |

| **Table S6.** Relative energies for interacted structures of proline conformers with five water molecules*^a^*,*^b^*,*^c^* | | | | |
| --- | --- | --- | --- | --- |
|  | **5W_I_** | | **5W_II_** | |
| **P_A_** | -0.5 | (-0.2) |  |  |
| **P_B_** | 0 | (0) | 2.4 | (2.0) |
| **P_C_** | -6.0 | (-10.5) | -4.0 | (-7.5) |
| *^a^* Energy units in kcal/mol;  *^b^* **P_B_5W_I_** as the energy benchmark;  *^c^* Solvent effects considered by the PCM model are given in parentheses. | | | | |

N


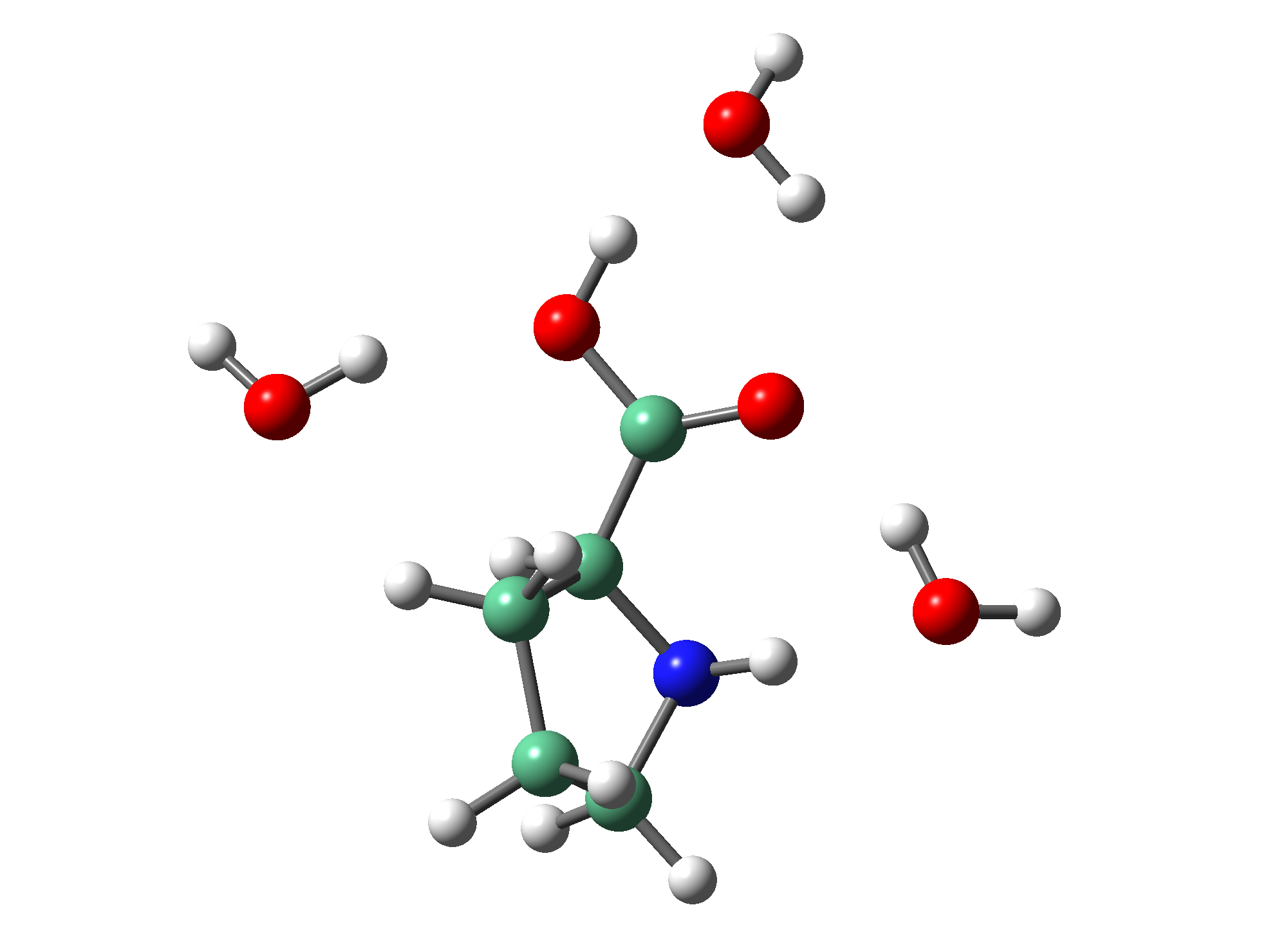

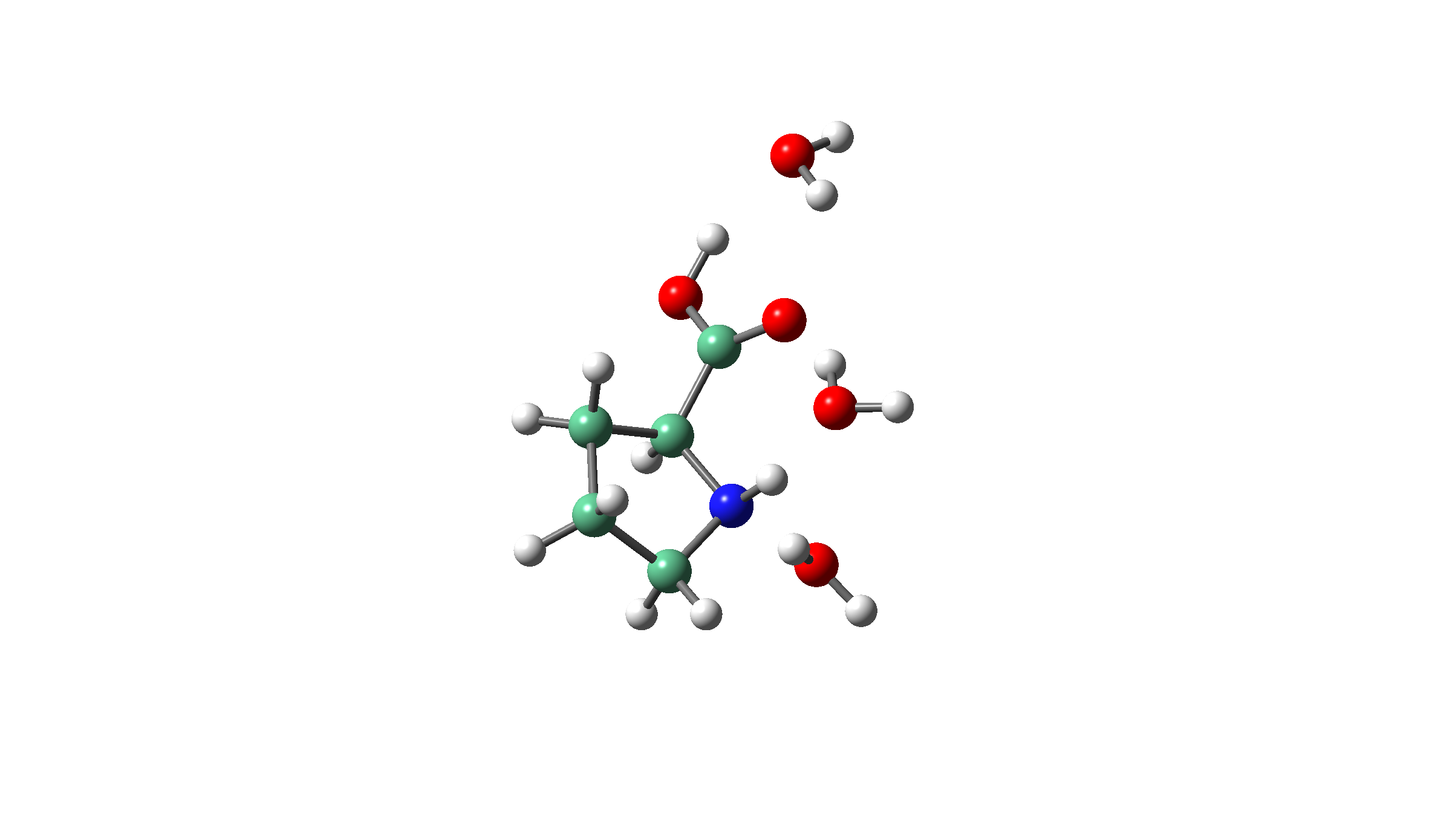

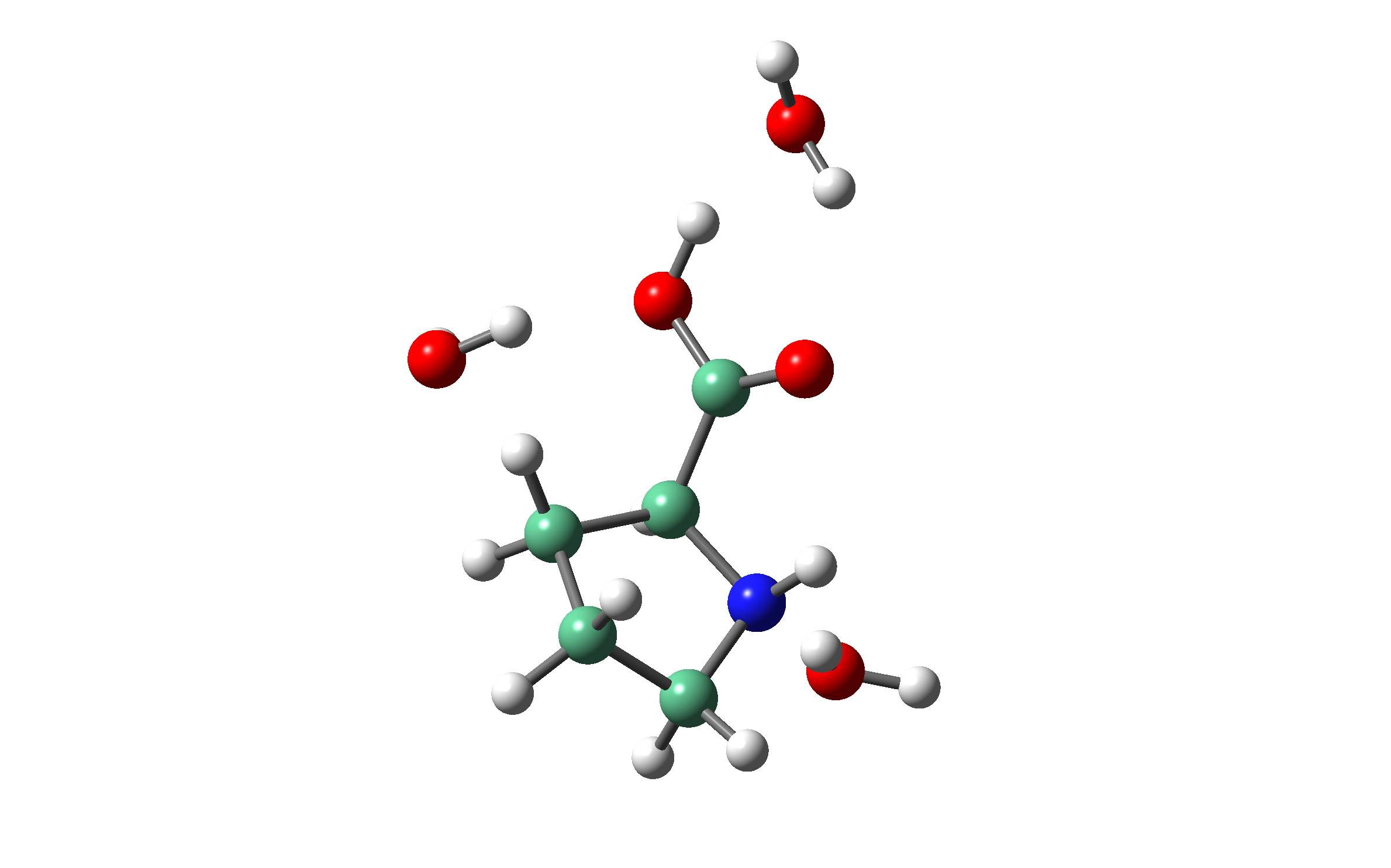

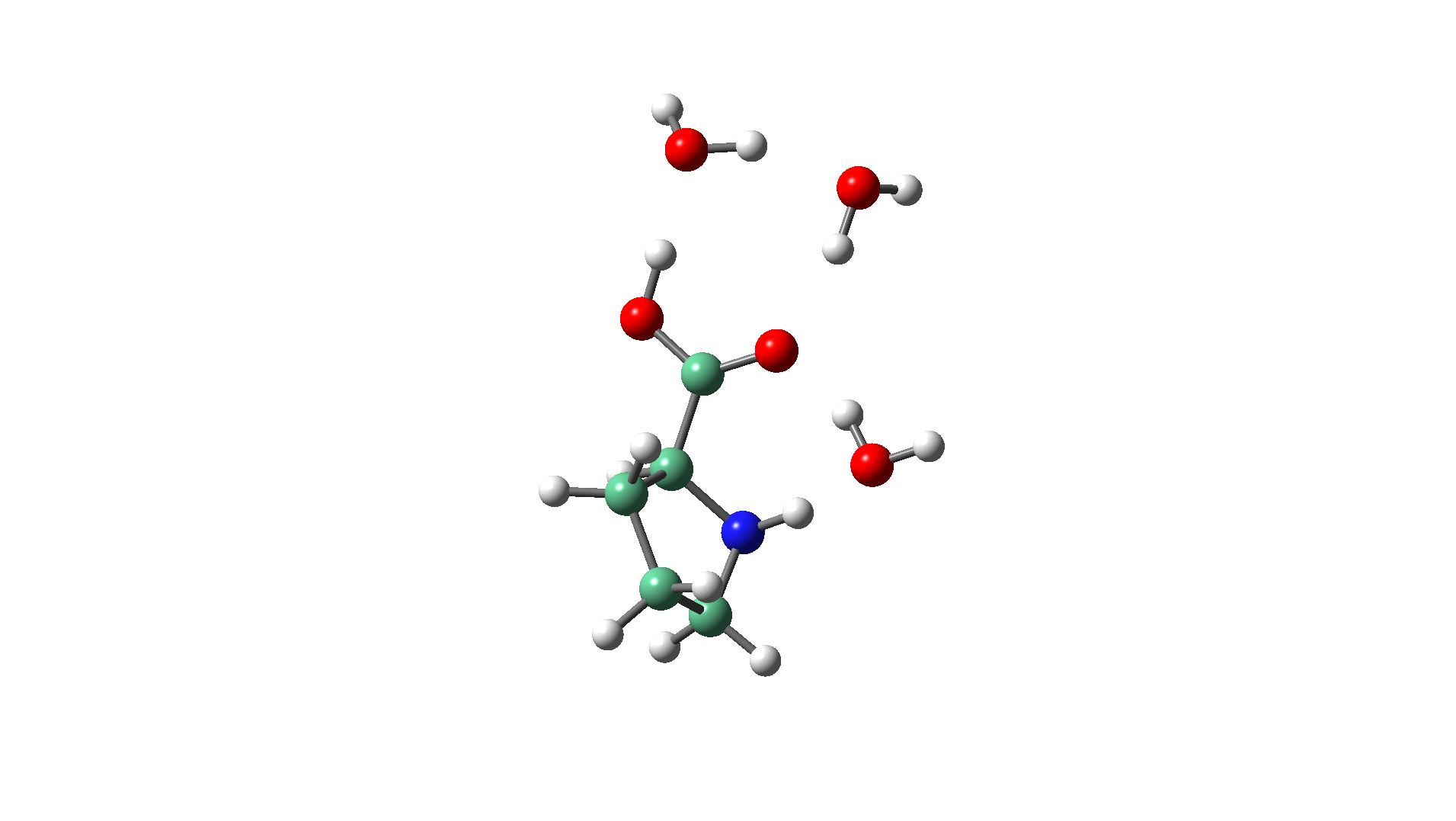

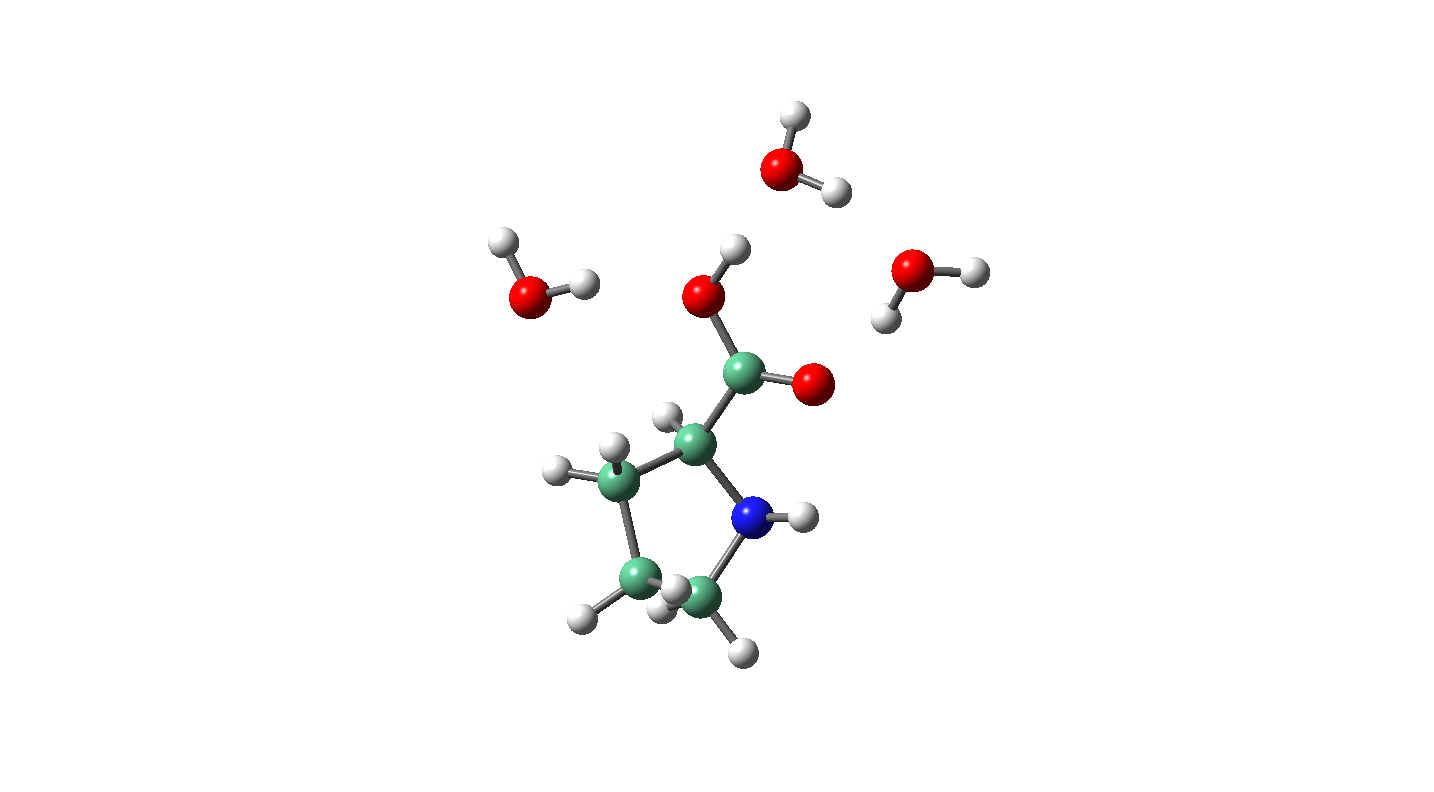


O1

O3

O4

O5

O2

N

O1

O3

O4

O5

O2

N

O1

O3

O4

O5

O2

O1

O3

O4

O5

O2

N

O1

O3

O4

O5

O2

N

(a) **P_A_3W_II_** (2.8) (b) **P_A_3W_III_** (3.7)

(c) **P_A_3W_IV_** (5.8) (d) **P_A_3W_V_** (7.6)

(e) **P_A_3W_VI_** (9.8)

**Figure S1.** Interacted structures of **P_A_** and three water molecules. Relative energies (kcal/mol) are given in parentheses, using **P_B_3W_I_** as benchmark. H-bonds are marked with dashed lines.

(a) **P_B_3W_II_** (0.4) (b) **P_B_3W_III_** (3.2)


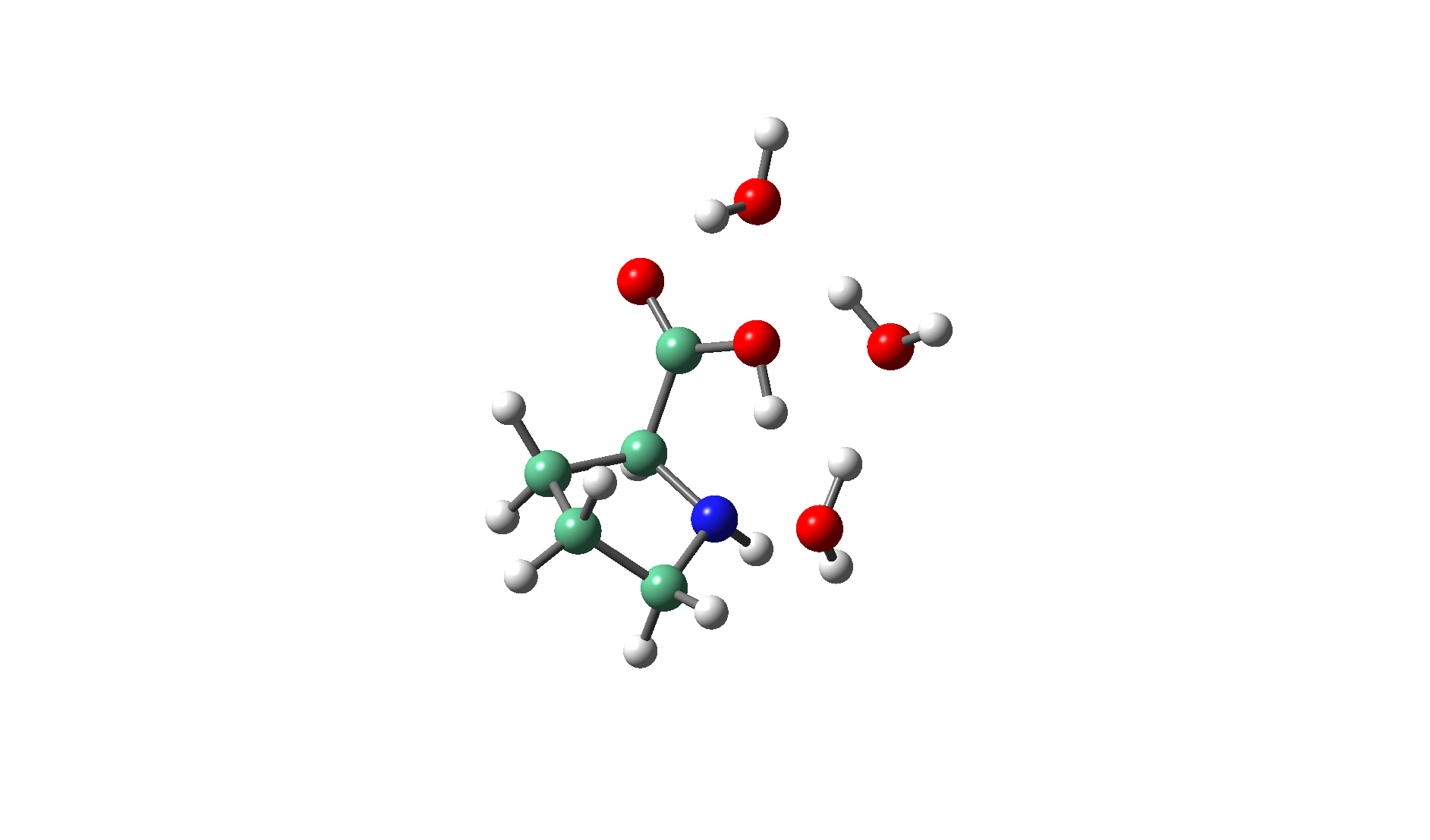


O2

O3

O4

O5

O1

N


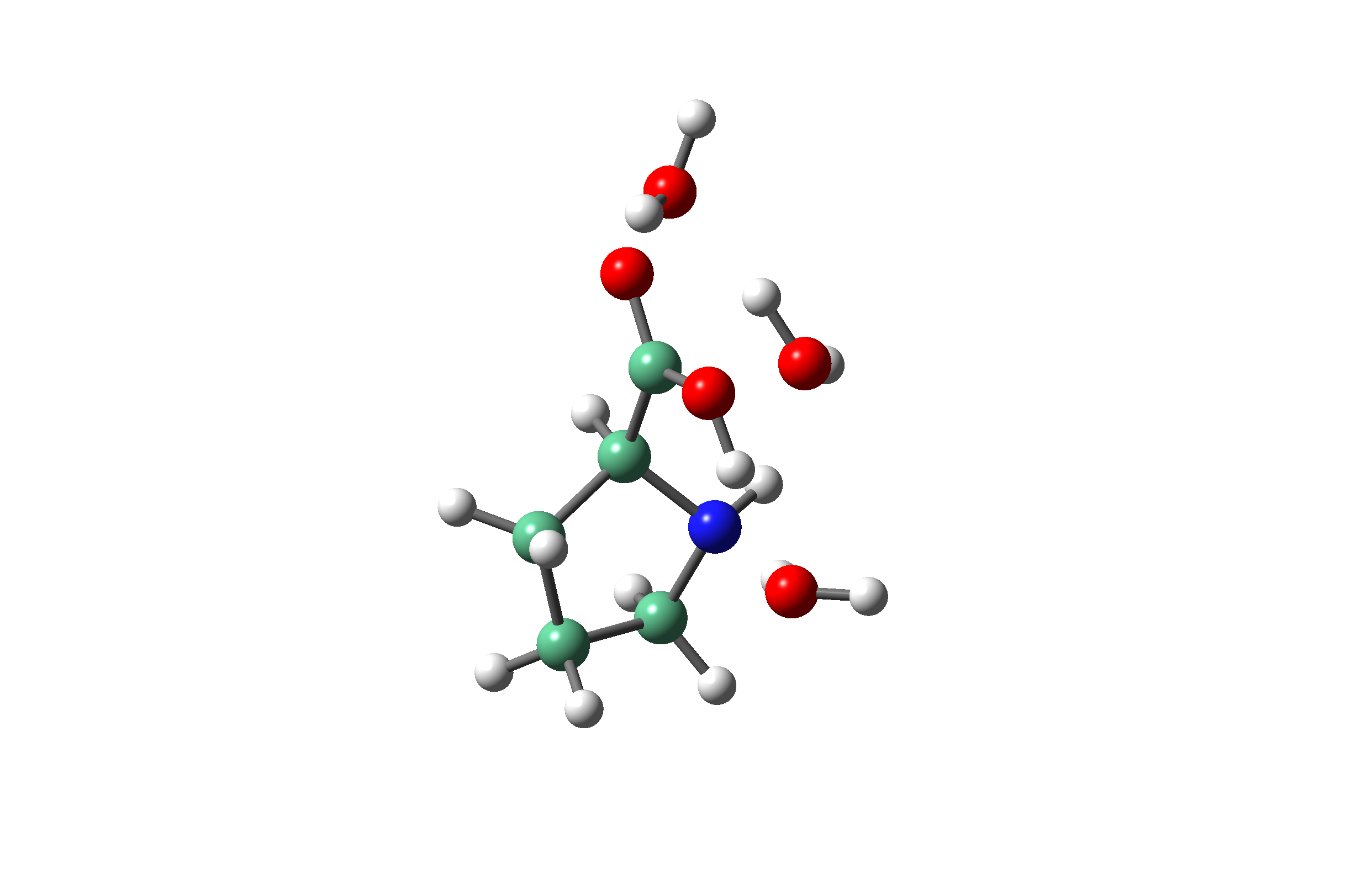


O1

O3

O5

O4

O2

N


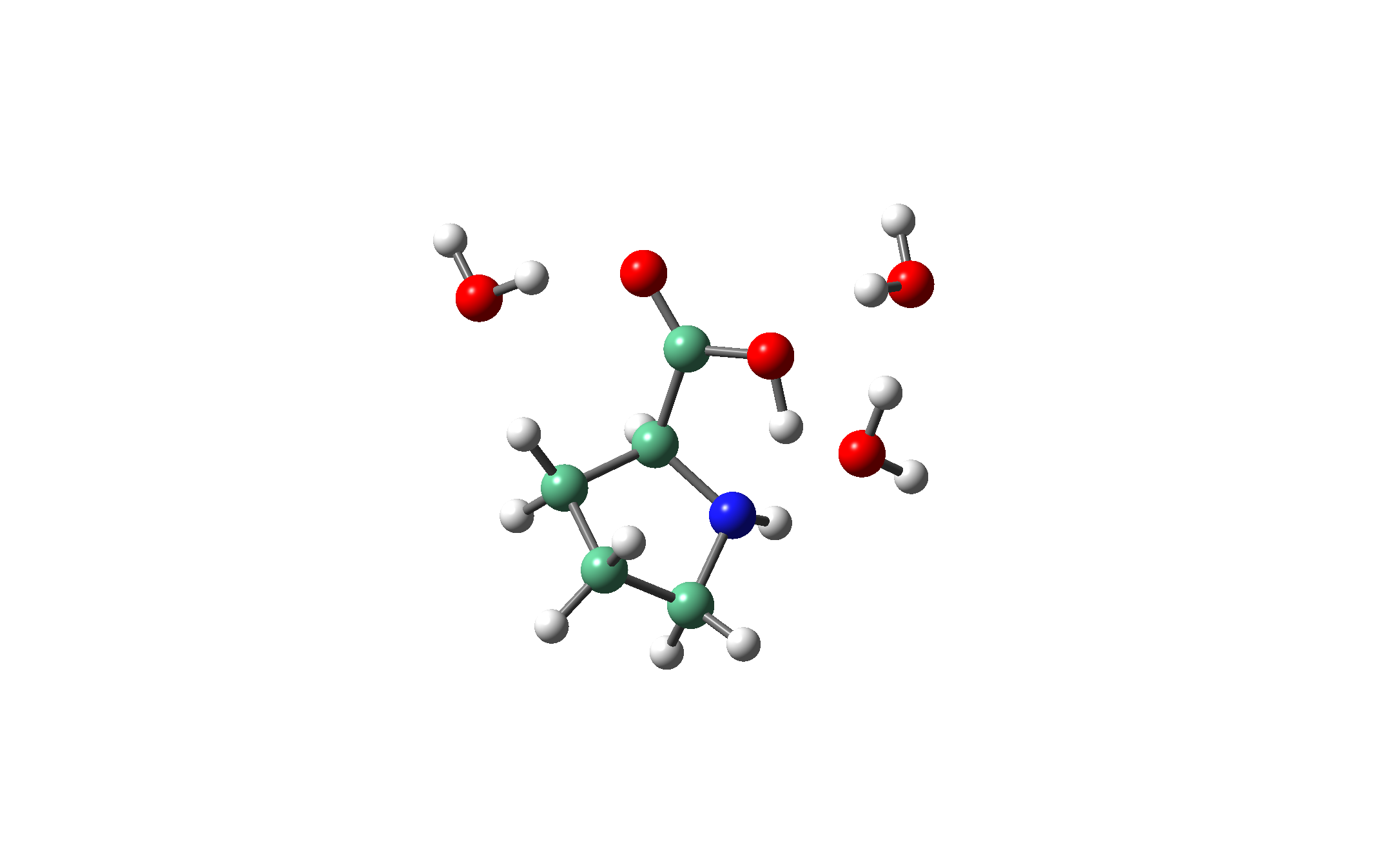


O2

O5

O3

O4

O1

N

(c) **P_B_3W_IV_** (5.2)

**Figure S2.** Interacted structures of **P_B_** and three water molecules. Relative energies (kcal/mol) are given in parentheses, using **P_B_3W_I_** as benchmark. H-bonds are marked with dashed lines.

(a) **P_C_3W_II_** (1.9) (b) **P_C_3W_III_** (2.1)

(c) **P_C_3W_IV_** (3.9) (d) **P_C_3W_V_** (4.1)


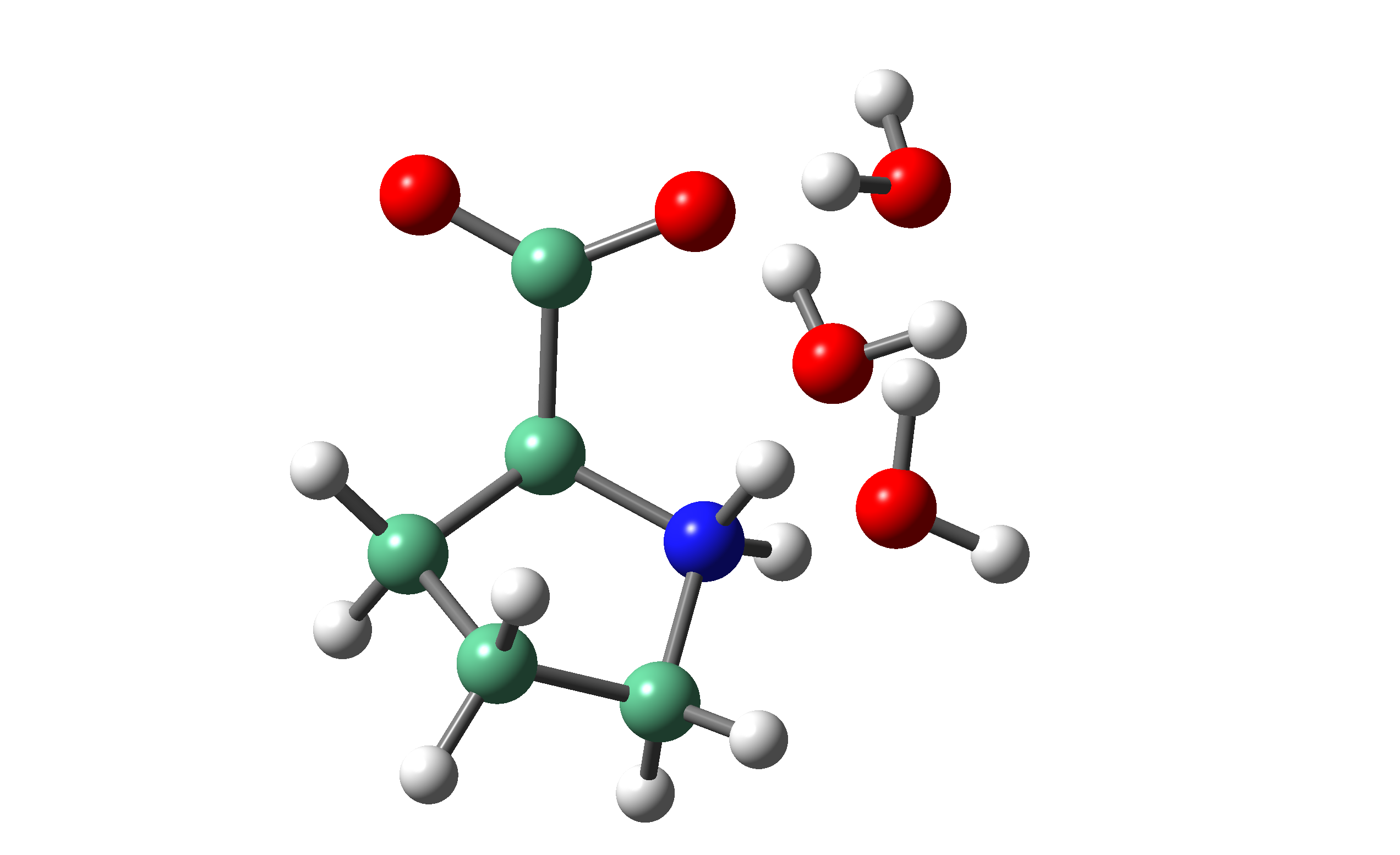

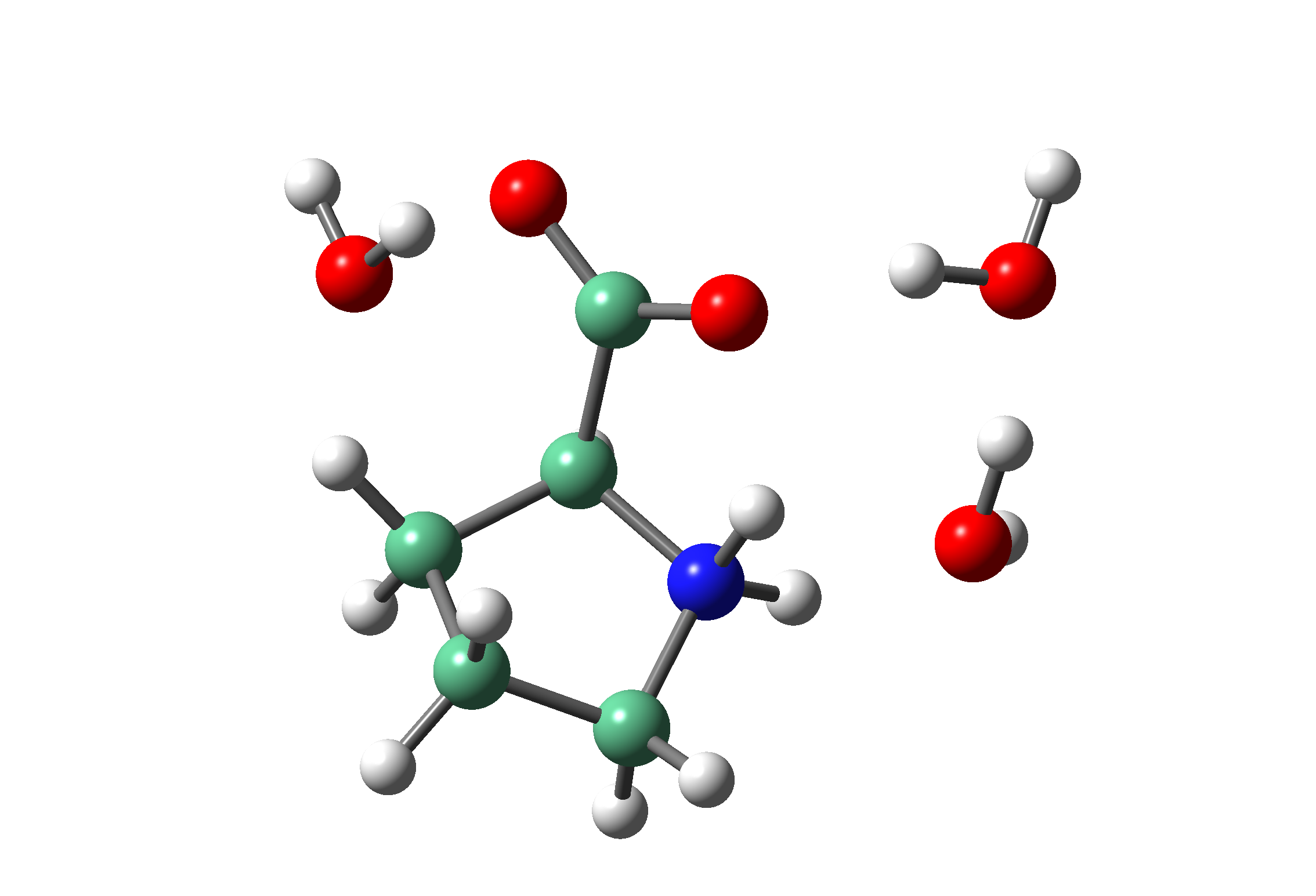


O1

O3

O5

O5

O2

N

O1

O3

O4

O2

N

O4


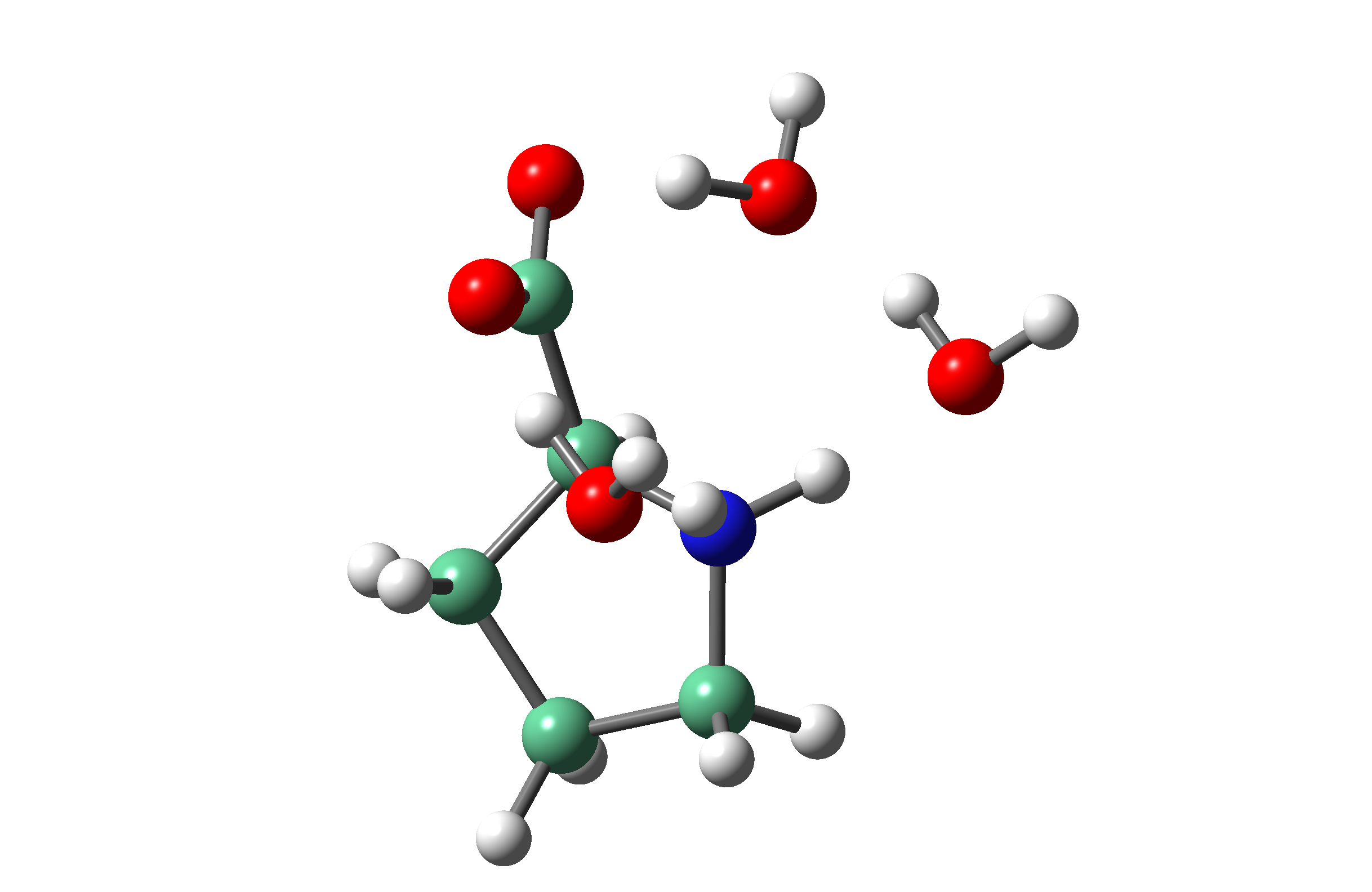

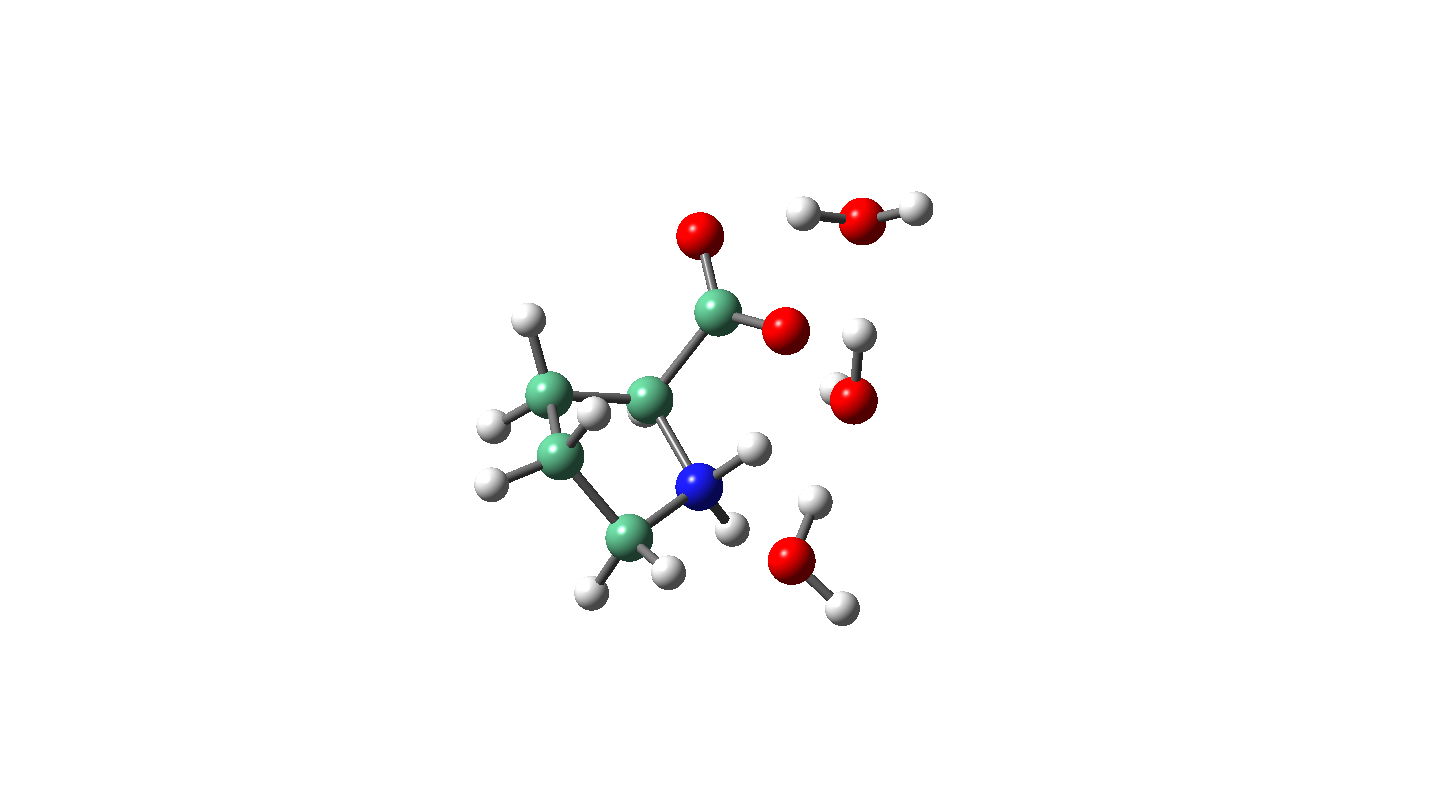


O1

O3

O5

O5

O2

N

O1

O3

O4

O2

N

O4

**Figure S3.** Interacted structures of **P_C_** and three water molecules. Relative energies (kcal/mol) are given in parentheses, using **P_B_3W_I_** as benchmark. H-bonds are marked with dashed lines.

(a) **P_A_4W_II_** (4.3) (b) **P_A_4W_III_** (7.2)


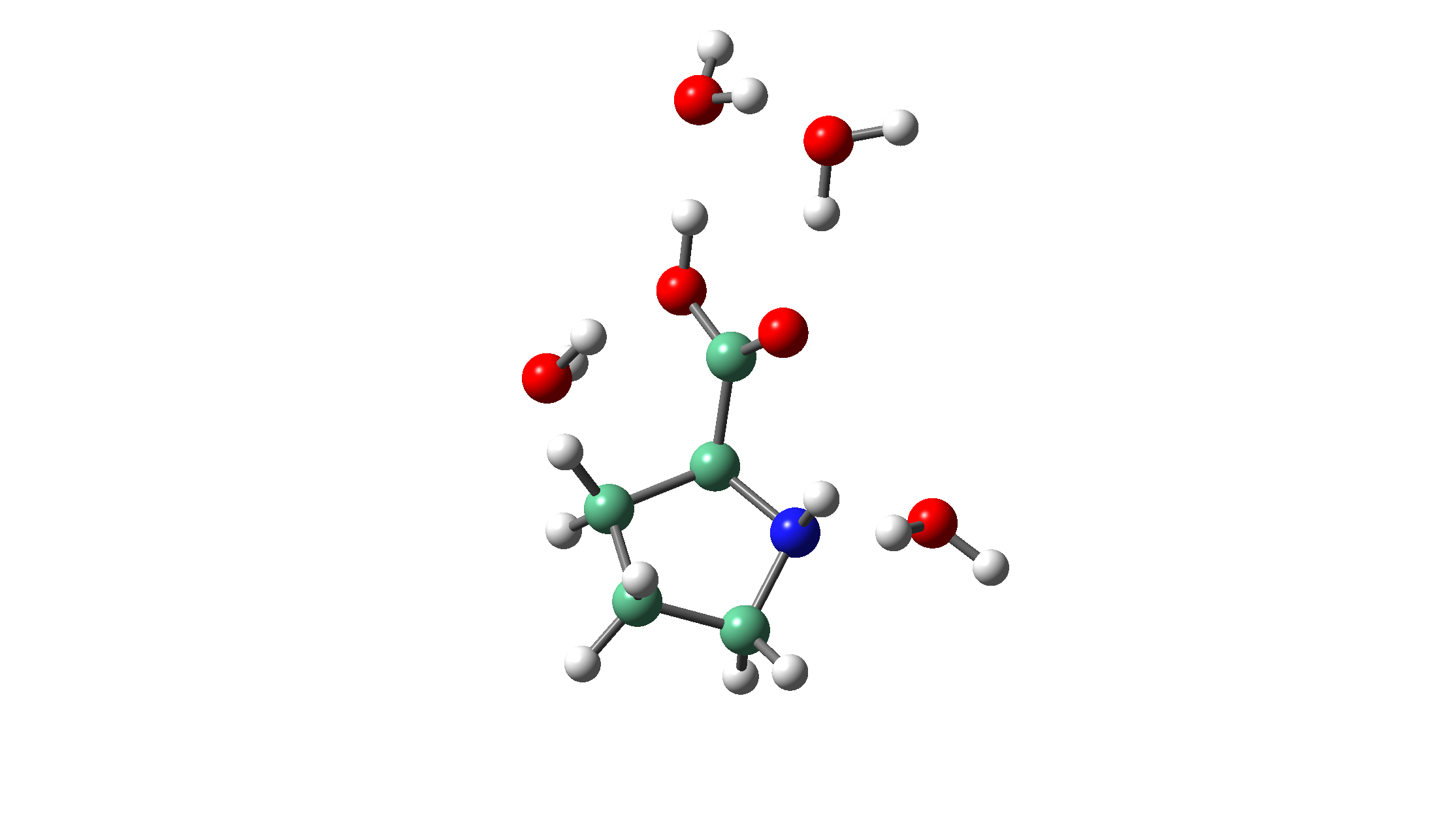

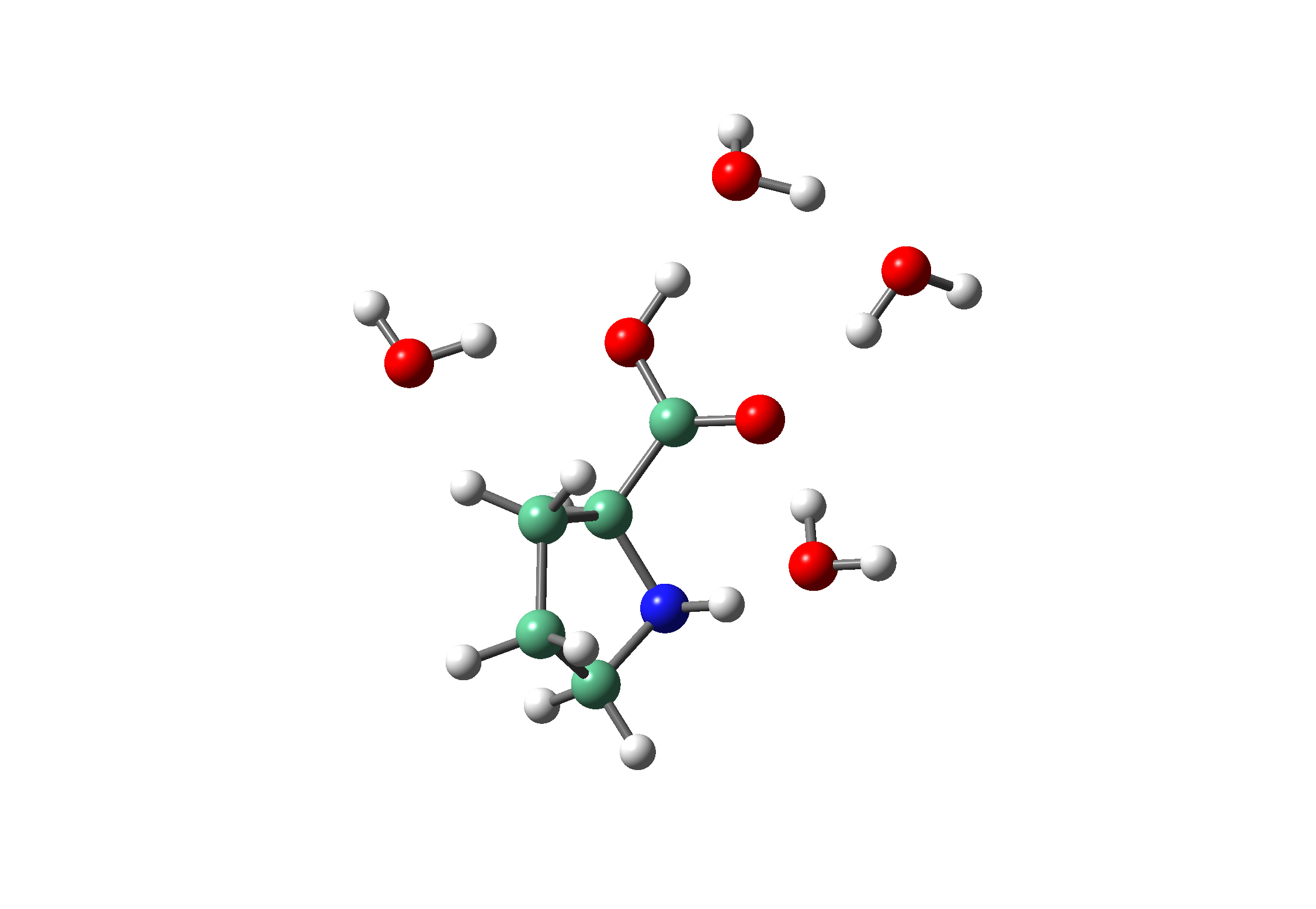


O1

O3

O4

O5

O2

N

O1

O3

O4

O5

O2

N

O6

O6

**Figure S4.** Interacted structures of **P_A_** and four water molecules. Relative energies (kcal/mol) are given in parentheses, using **P_B_4W_I_** as benchmark. H-bonds are marked with dashed lines.


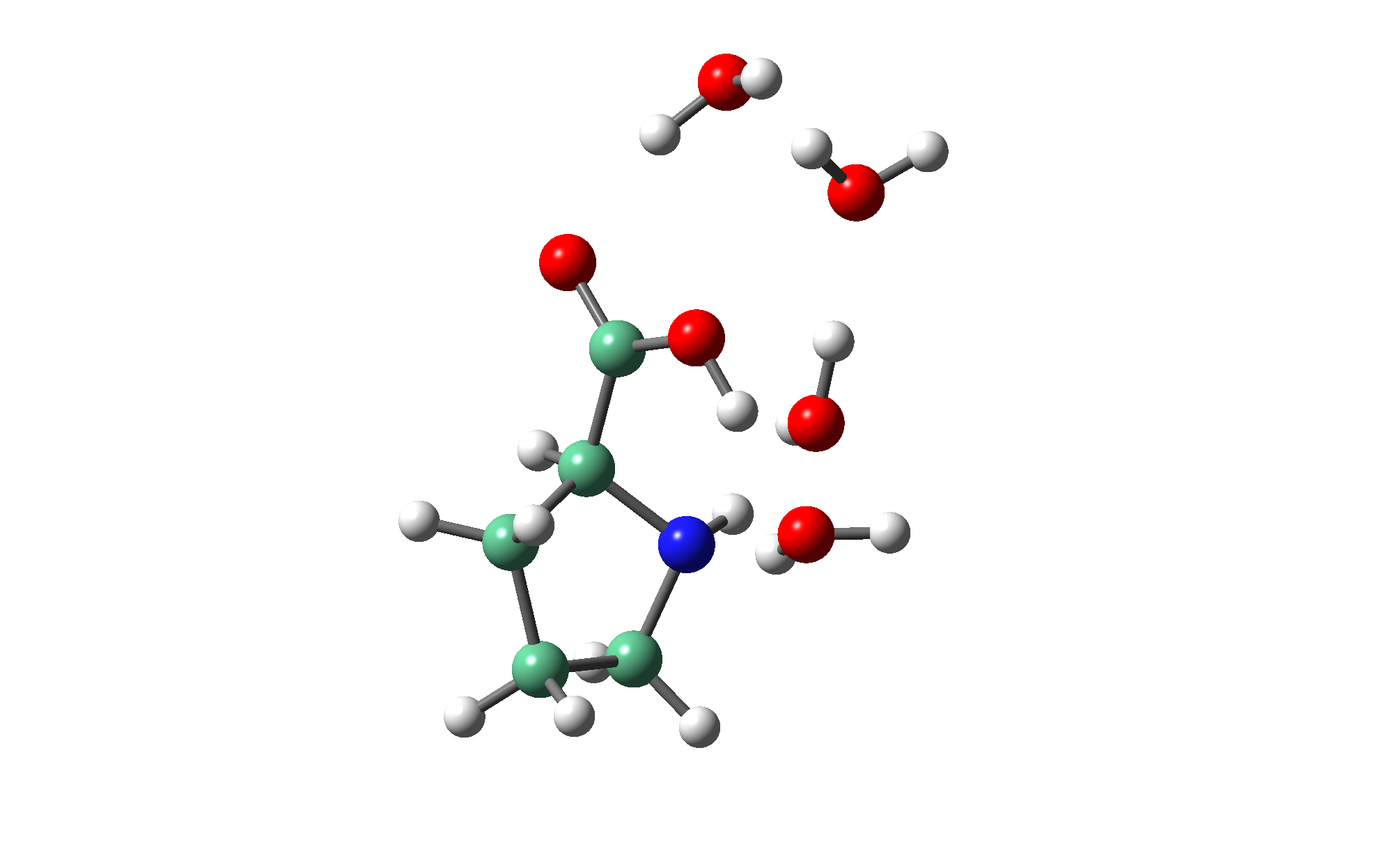

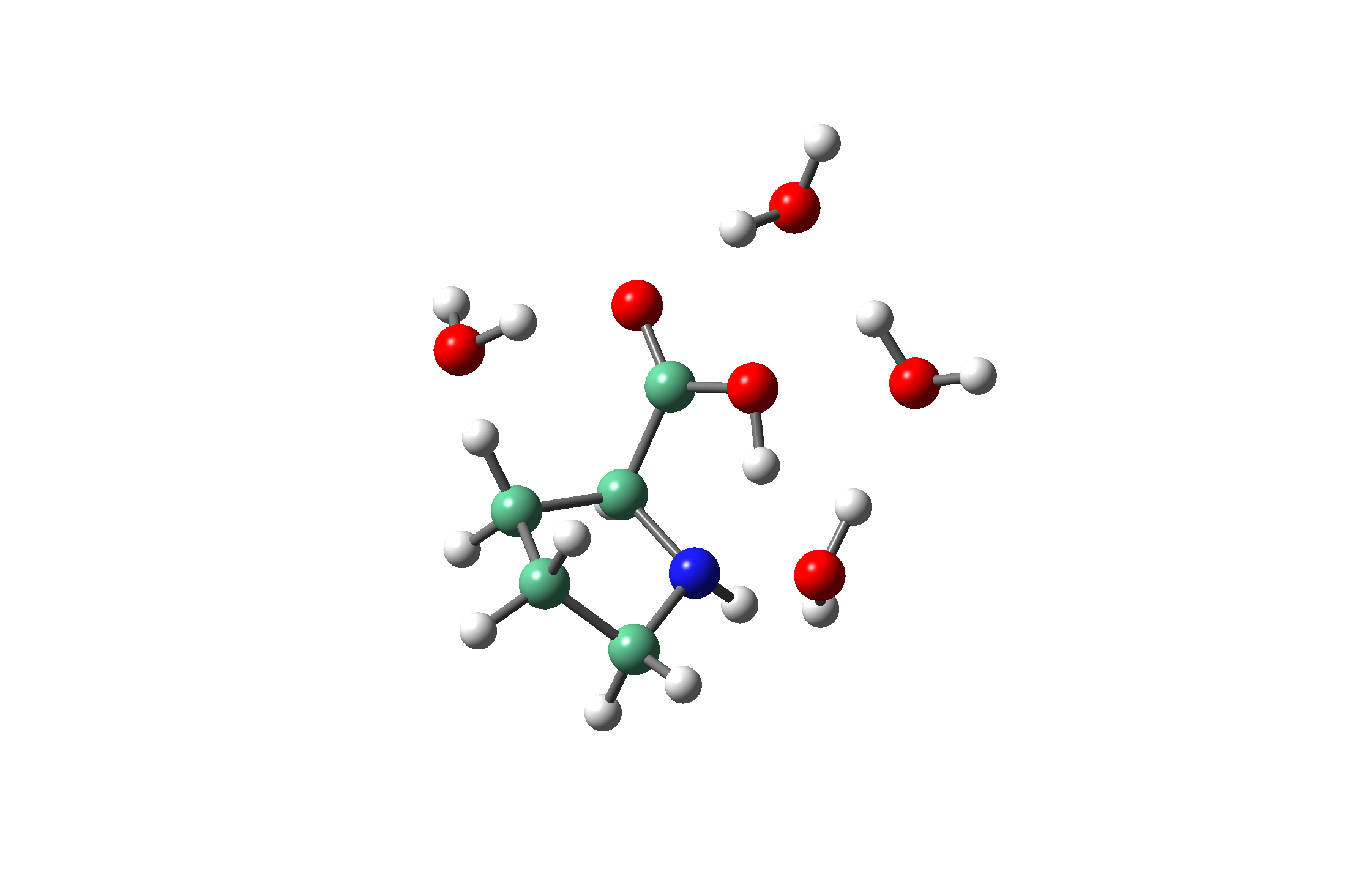


(a) **P_B_4W_II_** (2.1) (b) **P_B_4W_III_** (2.4)

O2

O3

O4

O5

O1

N

O1

O3

O4

O6

O2

N

O6

O5

**Figure S5.** Interacted structures of **P_B_** and four water molecules. Relative energies (kcal/mol) are given in parentheses, using **P_B_4W_I_** as benchmark. H-bonds are marked with dashed lines.


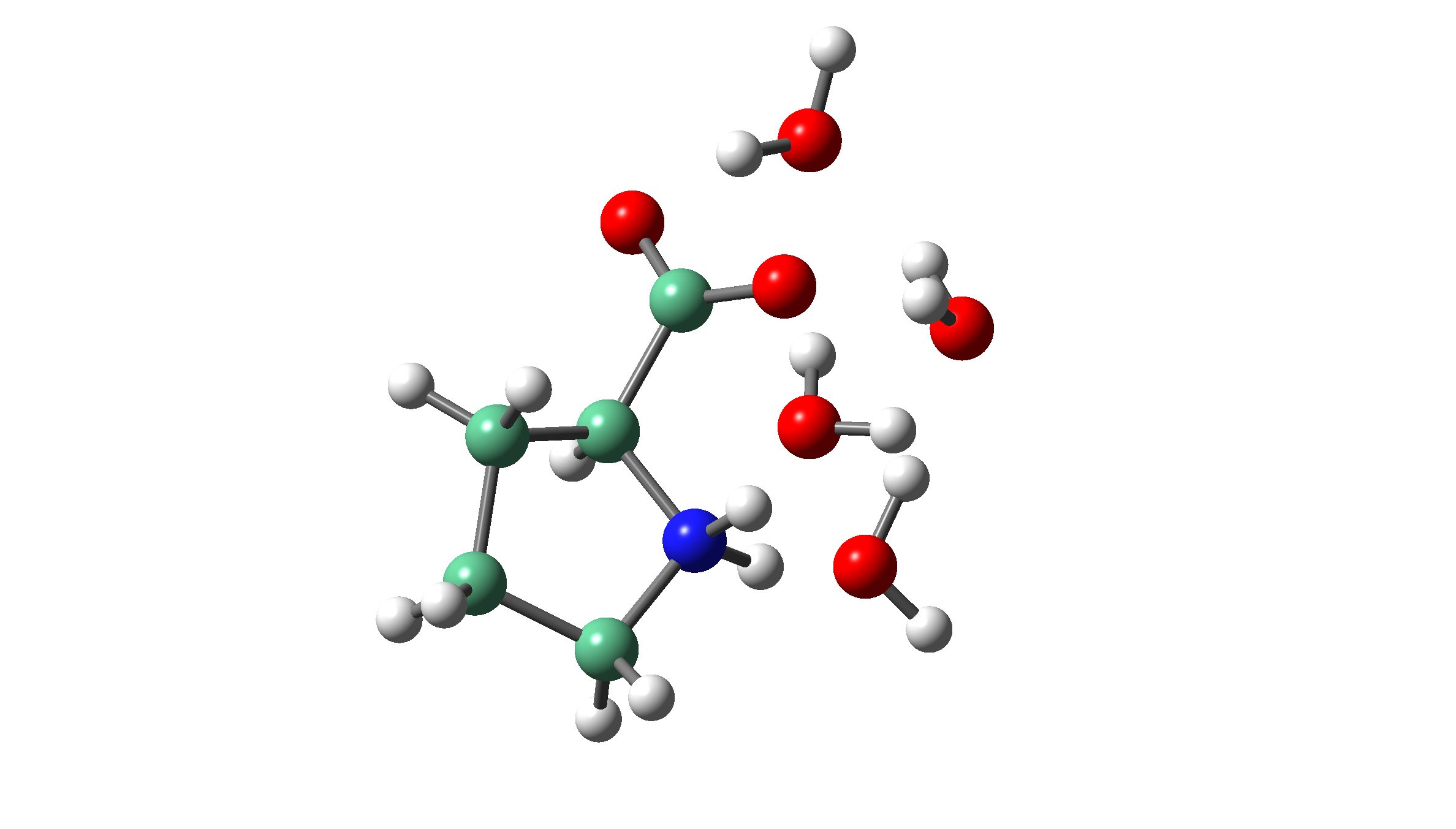

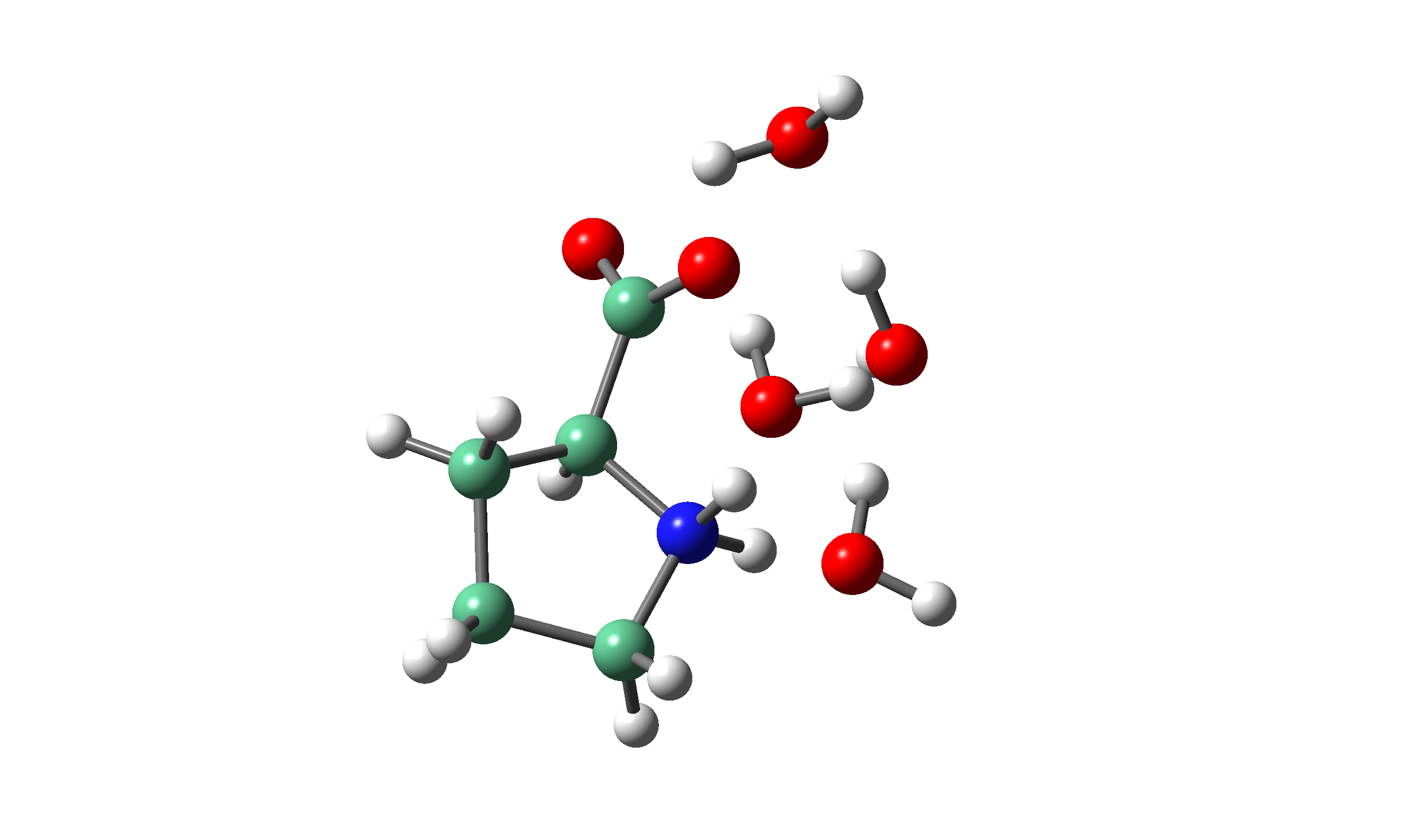

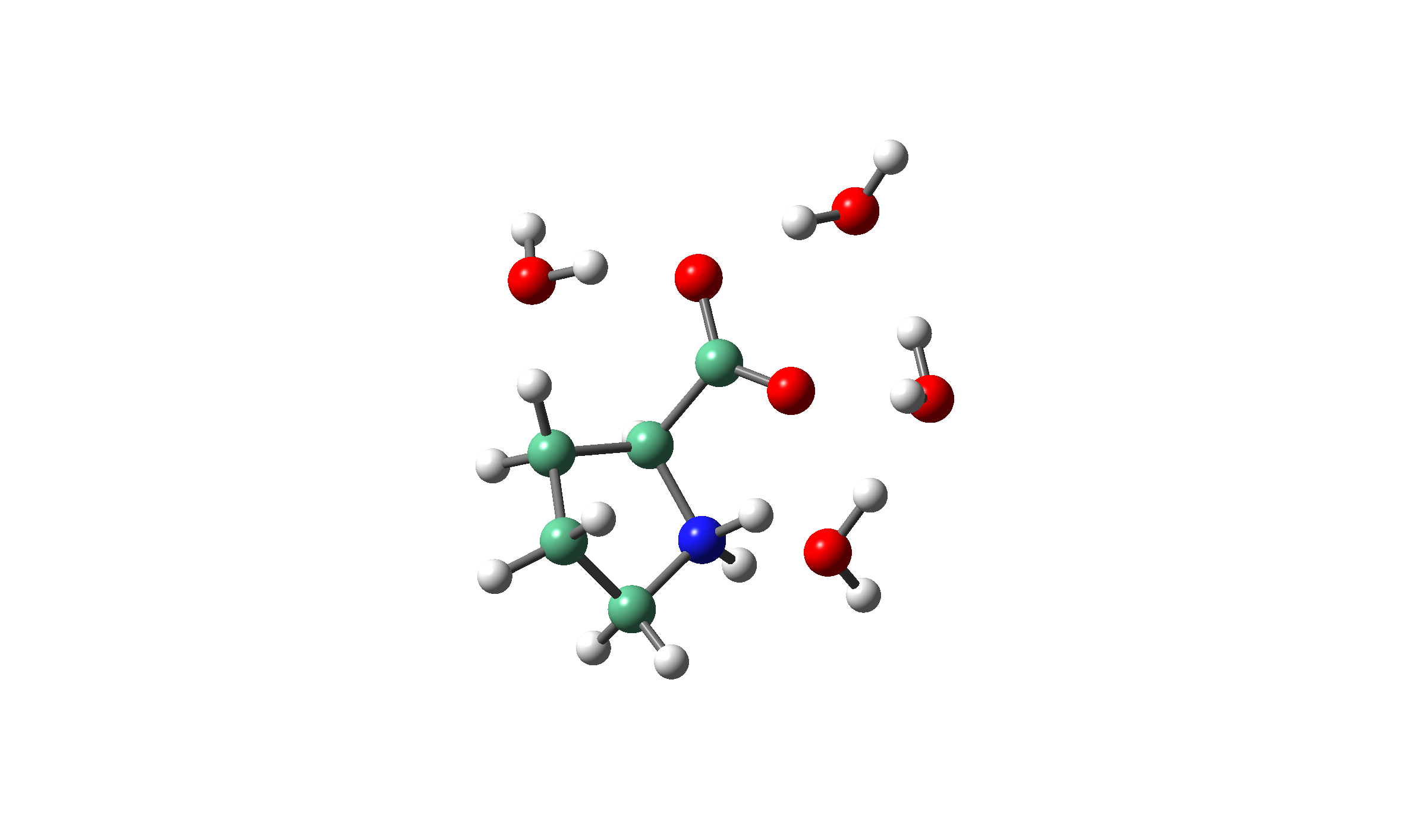


O1

O3

O4

O5

O6

N

O1

O3

O4

O5

O2

N

O2

O3

O5

O6

O1

N

O2

(a) **P_C_4W_II_** (-1.3) (b) **P_C_4W_III_** (-0.3)

(c) **P_C_4W_IV_** (0.3)

O6

O4

**Figure S6.** Interacted structures of **P_C_** and four water molecules. Relative energies (kcal/mol) are given in parentheses, using **P_B_4W_I_** as benchmark. H-bonds are marked with dashed lines.


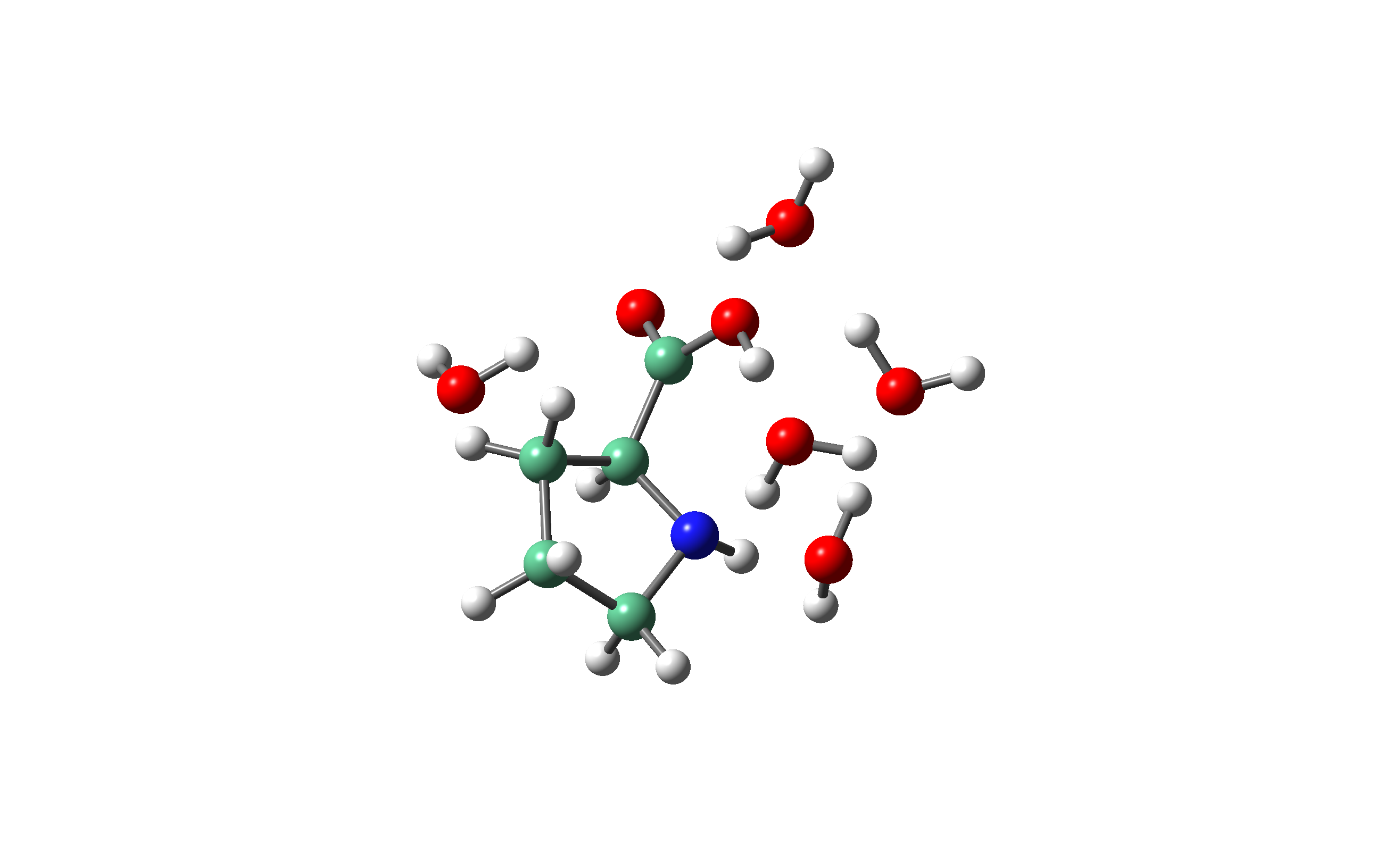


O6

O3

O4

O5

O2

N

O1

O7

**P_B_5W_II_** (2.4)

**Figure S7.** Interacted structure of **P_B_** and five water molecules (**P_B_5W_II_**). Relative energies (kcal/mol) are given in parentheses, using **P_B_5W_I_** as benchmark. H-bonds are marked with dashed lines.


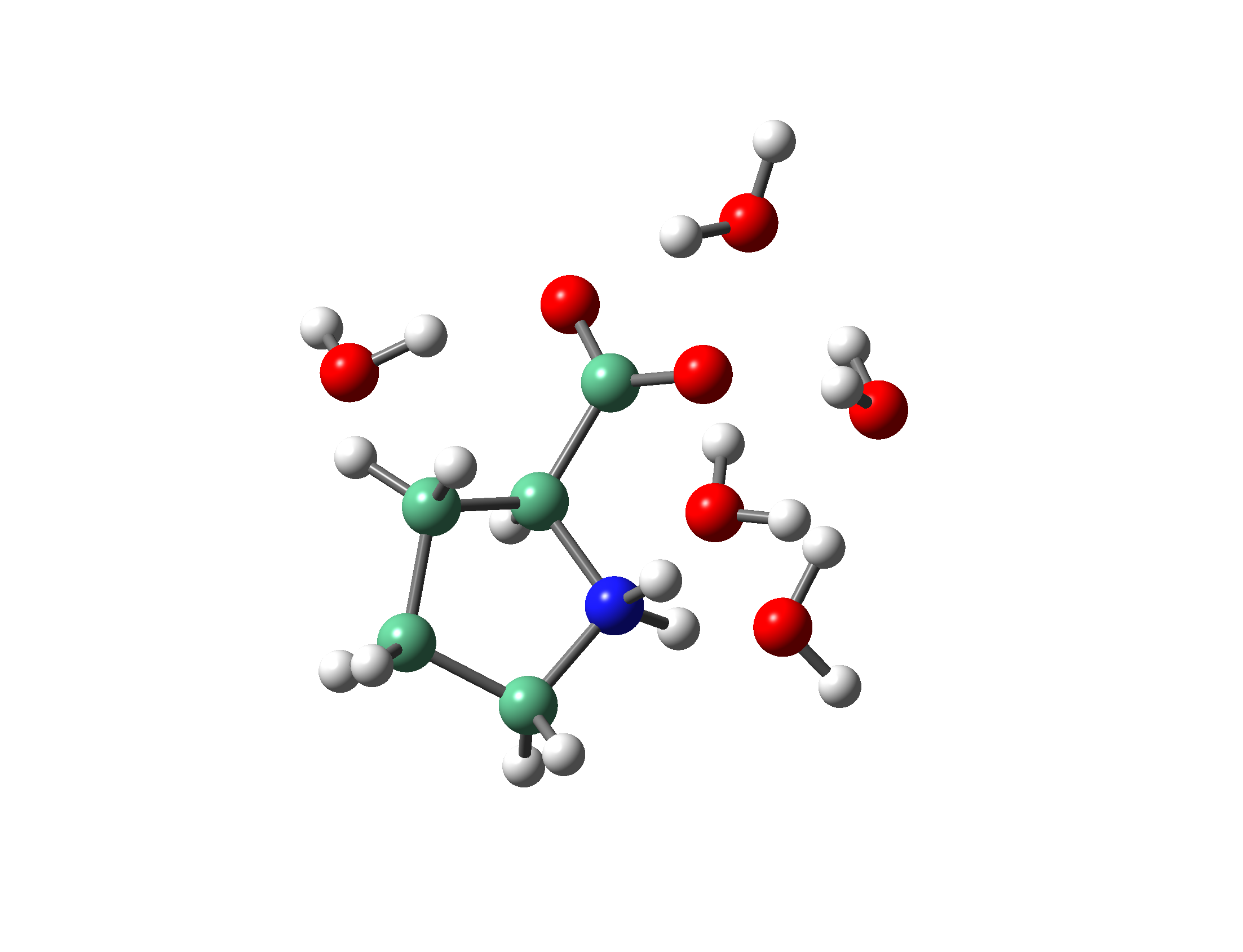


O1

O3

O4

O5

O2

N

O7

O6

**P_C_5W_II_** (-4.0)

**Figure S8.** Interacted structure of **P_C_** and five water molecules (**P_C_5W_II_**). Relative energies (kcal/mol) are given in parentheses, using **P_B_5W_I_** as benchmark. H-bonds are marked with dashed lines.


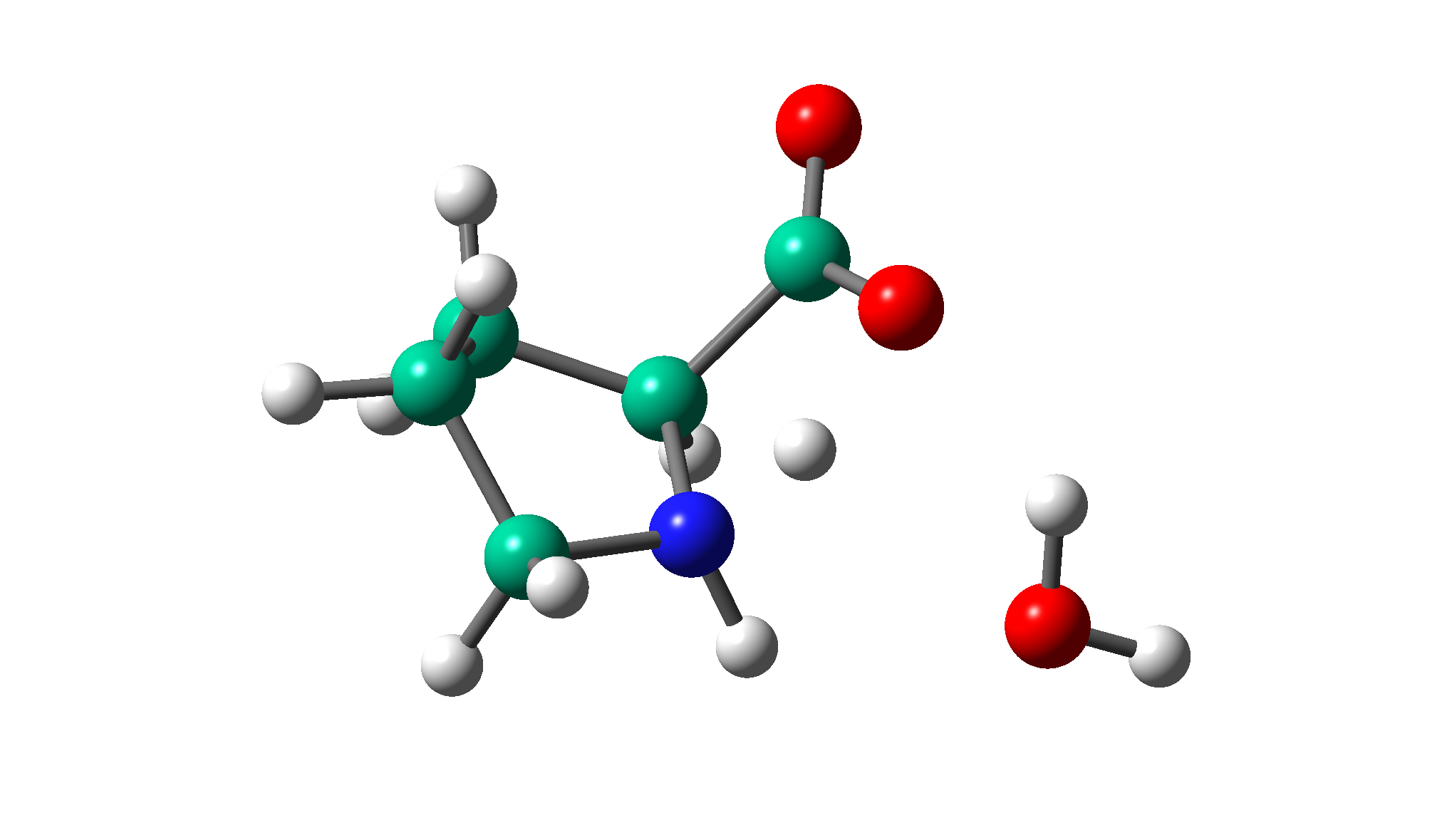


1.197

2.339

1.335

H3

O2

O1

O3

H2

N

H1

2.592

2.038

**TS1W_III-I_** (6.7)

**Figure S9.** Transition state structure for the conformational transformation from canonical to zwitterionic proline with presence of one water molecule (**TS1W_III-I_**). Activation barriers (kcal/mol) are given in parentheses. H-bonds (Å) are marked with dashed lines.

(a) **TS2W_II-I_** (4.2)  (b) **TS2W_I-II_** (6.1)

(c) **TS2W_VI-III_** (4.9) (d) **TS2W_III-IV_** (6.4)

(e) **TS2W_VII-V_** (6.2)  (f) **TS2W_V-VI_** (5.8)


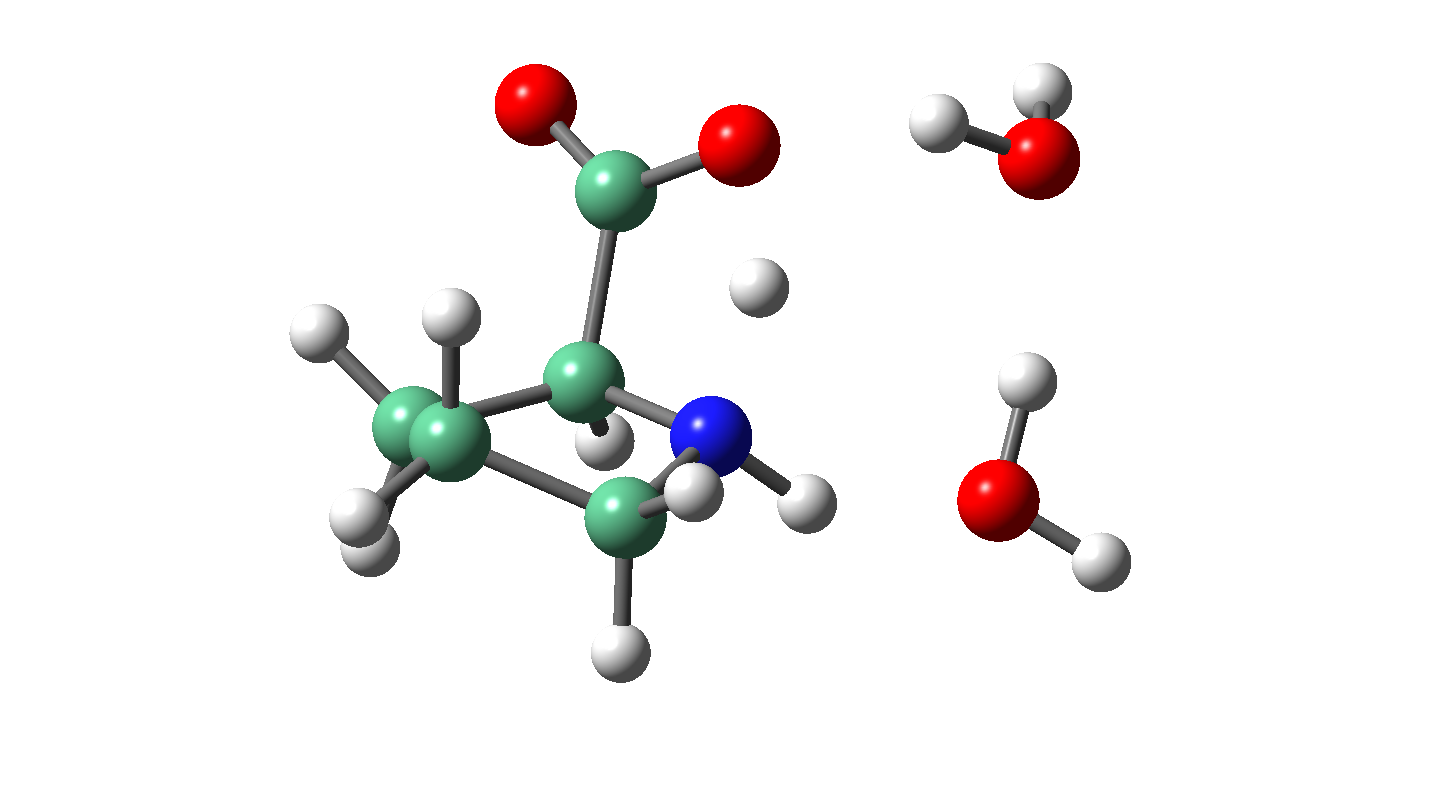


O4

O1

O3

1.846

H1

1.264

1.970

1.253

H2

1.795


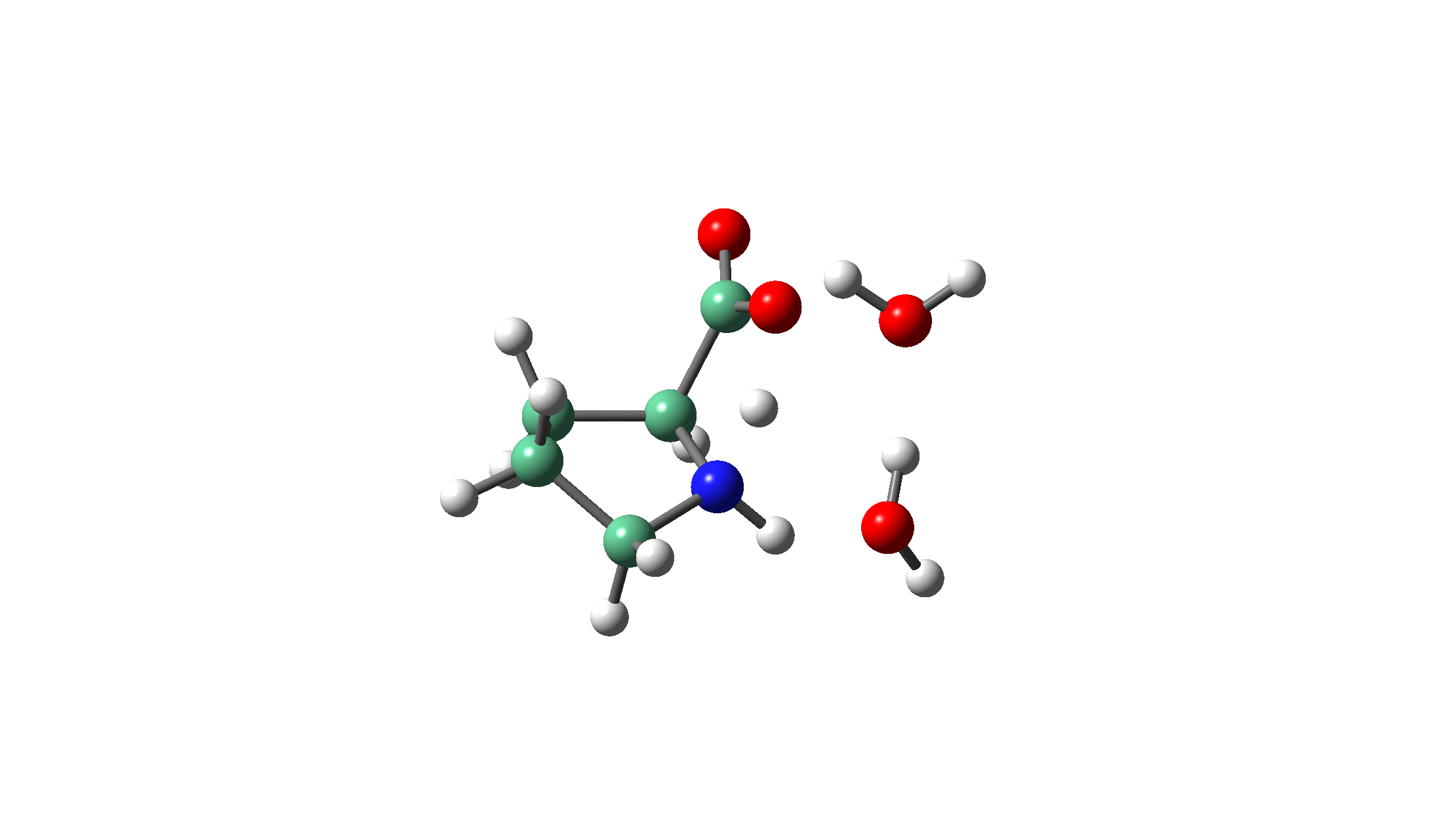


O4

O2

1.897

H1

1.292

1.814

1.237

H2

2.080

O3

O1


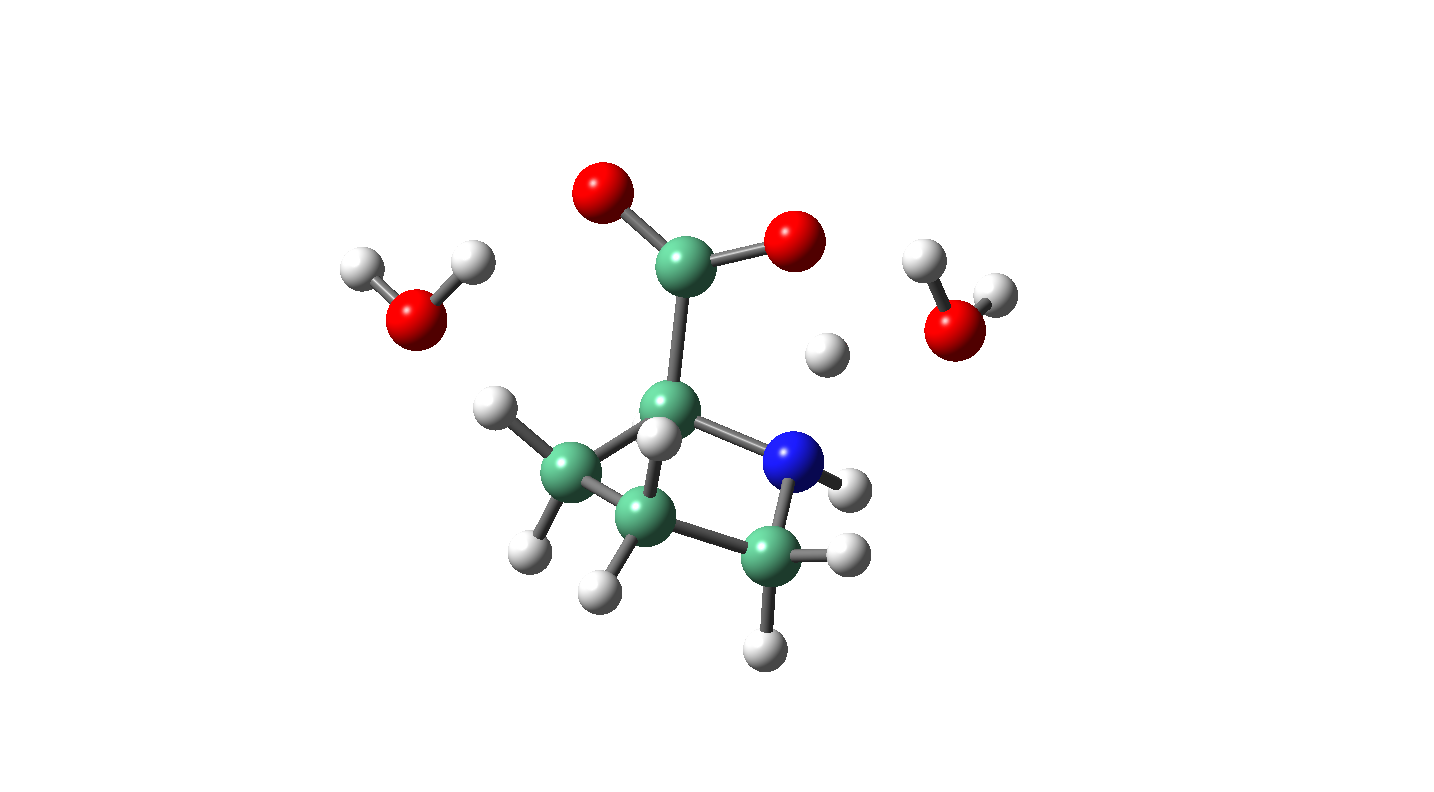


O2

1.830

H1

2.139

2.633

1.271

H2

2.341

O4

O3

H3

1.248


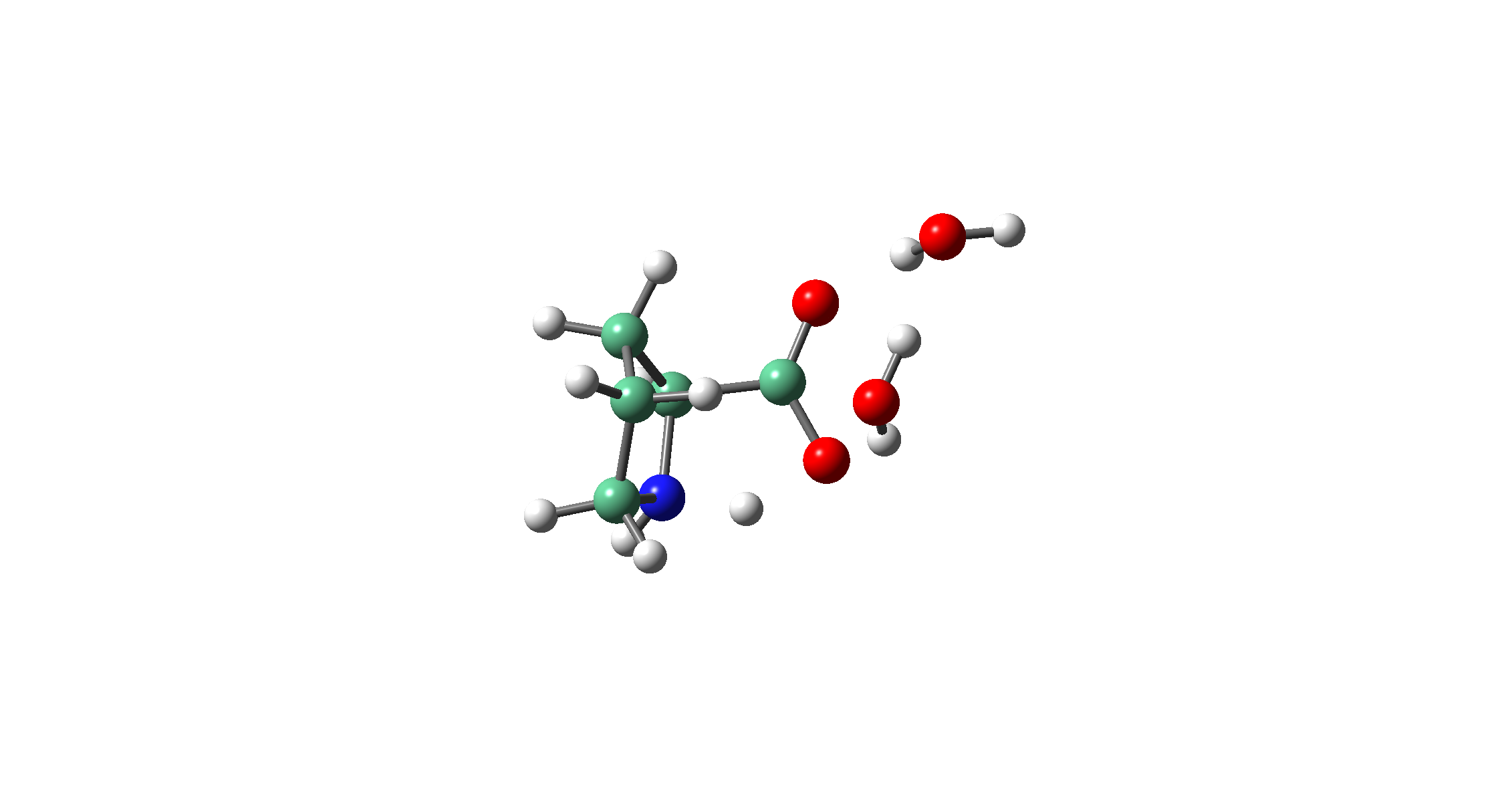


O2

1.842

1.945

1.339

1.195

O1

O3

H5

2.172

O4


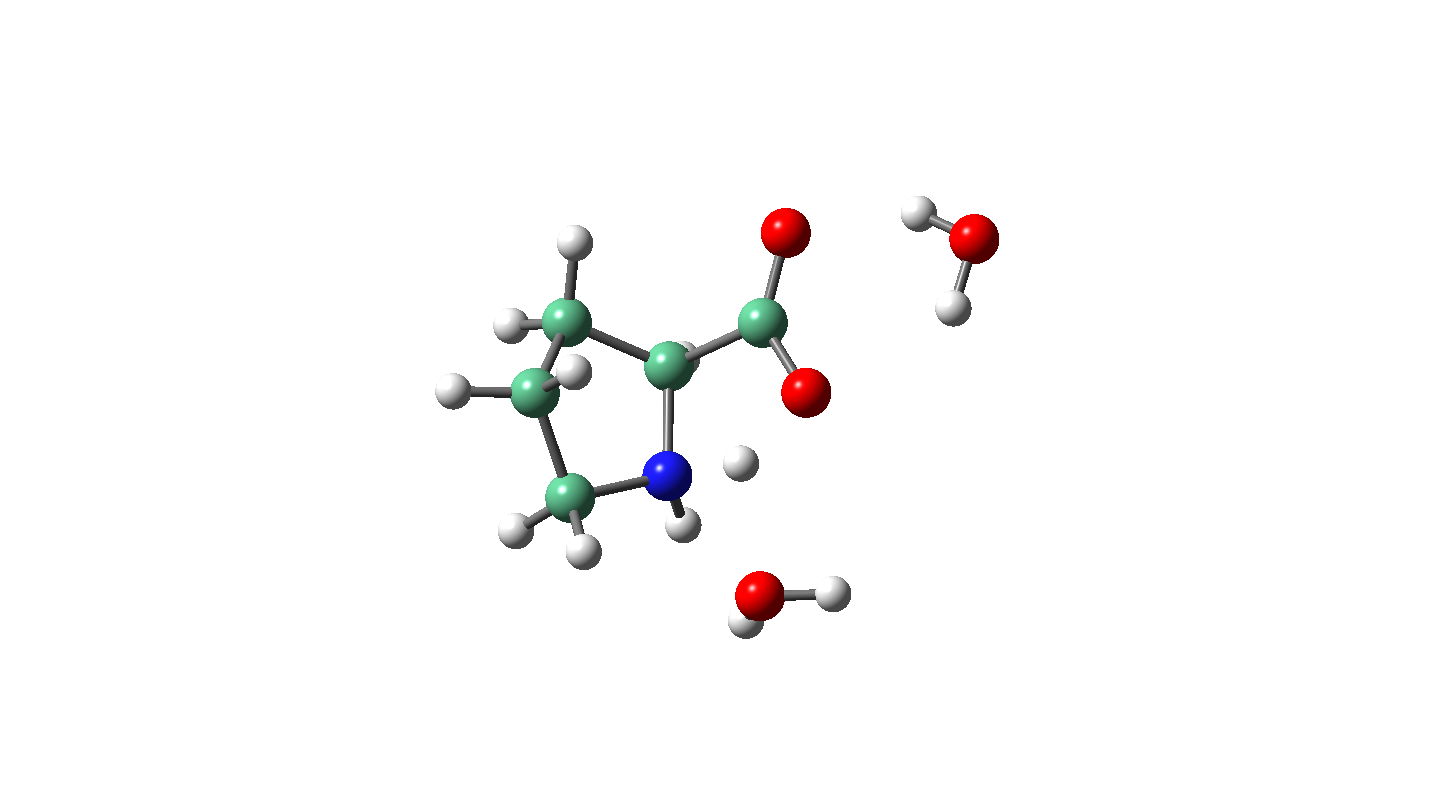


1.318

1.209

1.971

2.051

2.415

O3

H1

O2

O4

O1


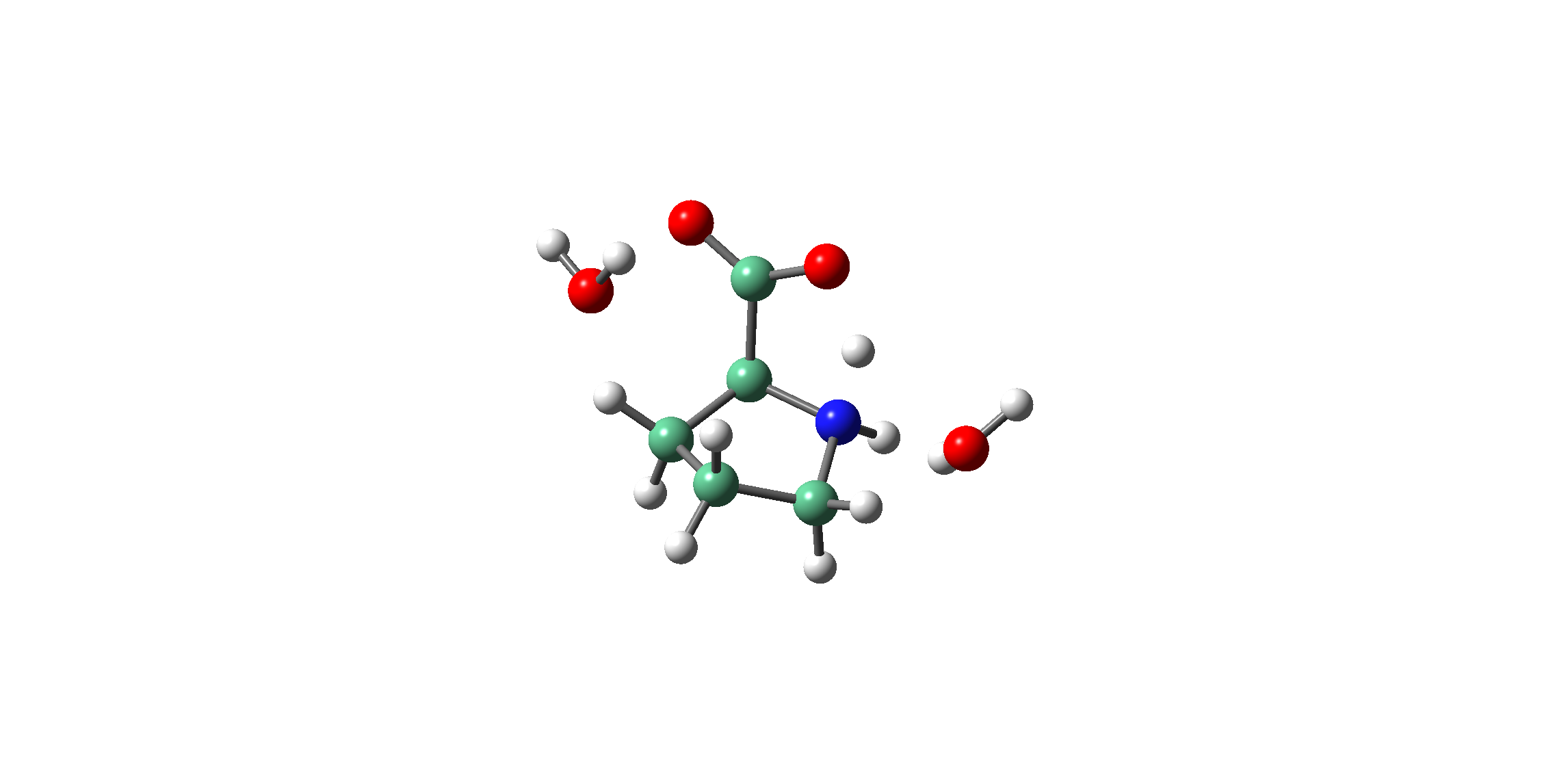


O2

1.805

1.317

1.963

O1

O3

H1

1.211

O4

(g) **TS2W_IV-VII_** (6.7)


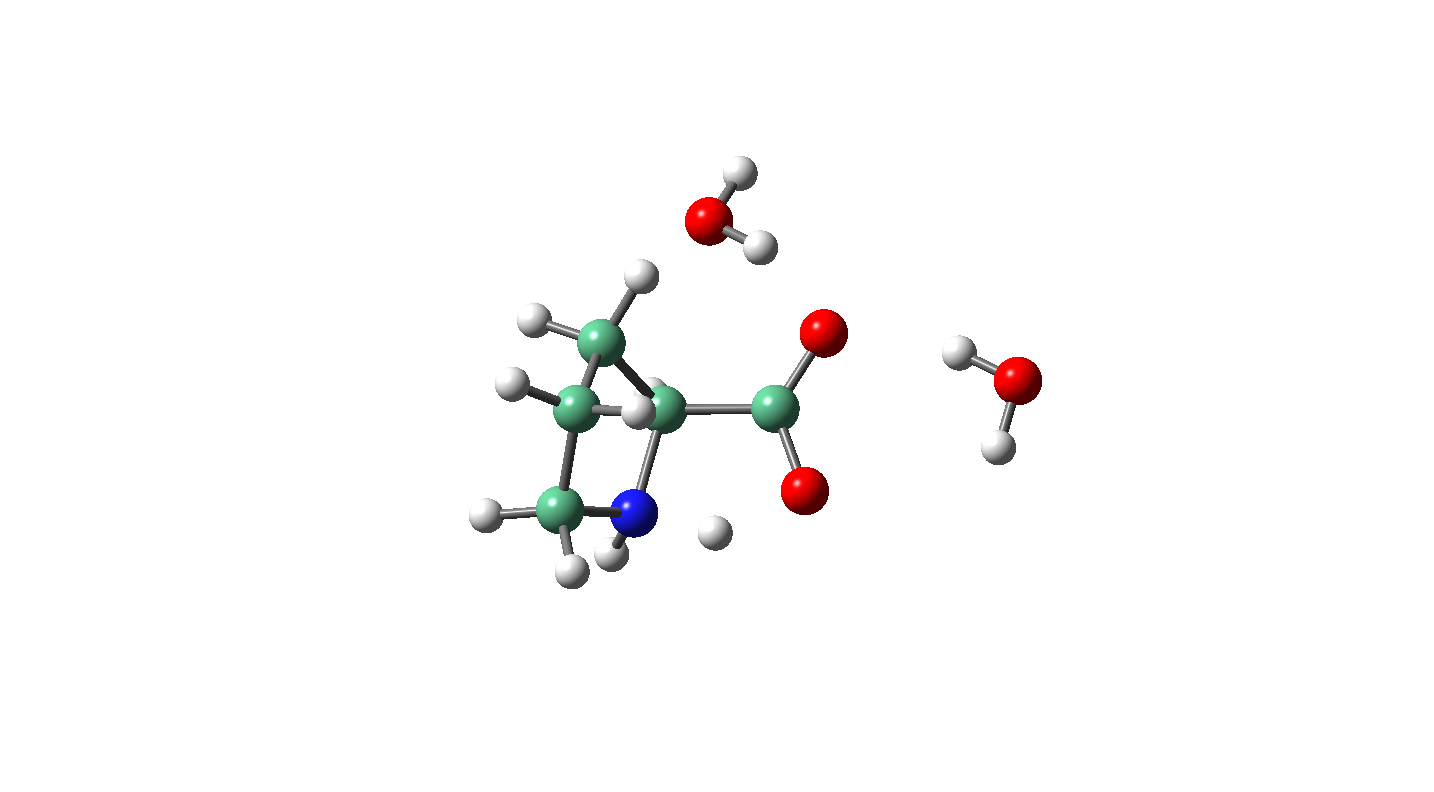


O2

2.766

H5

1.853

1.187

H2

1.352

O4

O3

H3

1.981

**Figure S10.** Transition state structures for the conformational transformations from canonical to zwitterionic proline with presence of two water molecules. Activation barriers (kcal/mol) are given in parentheses. H-bonds (Å) are marked with dashed lines.


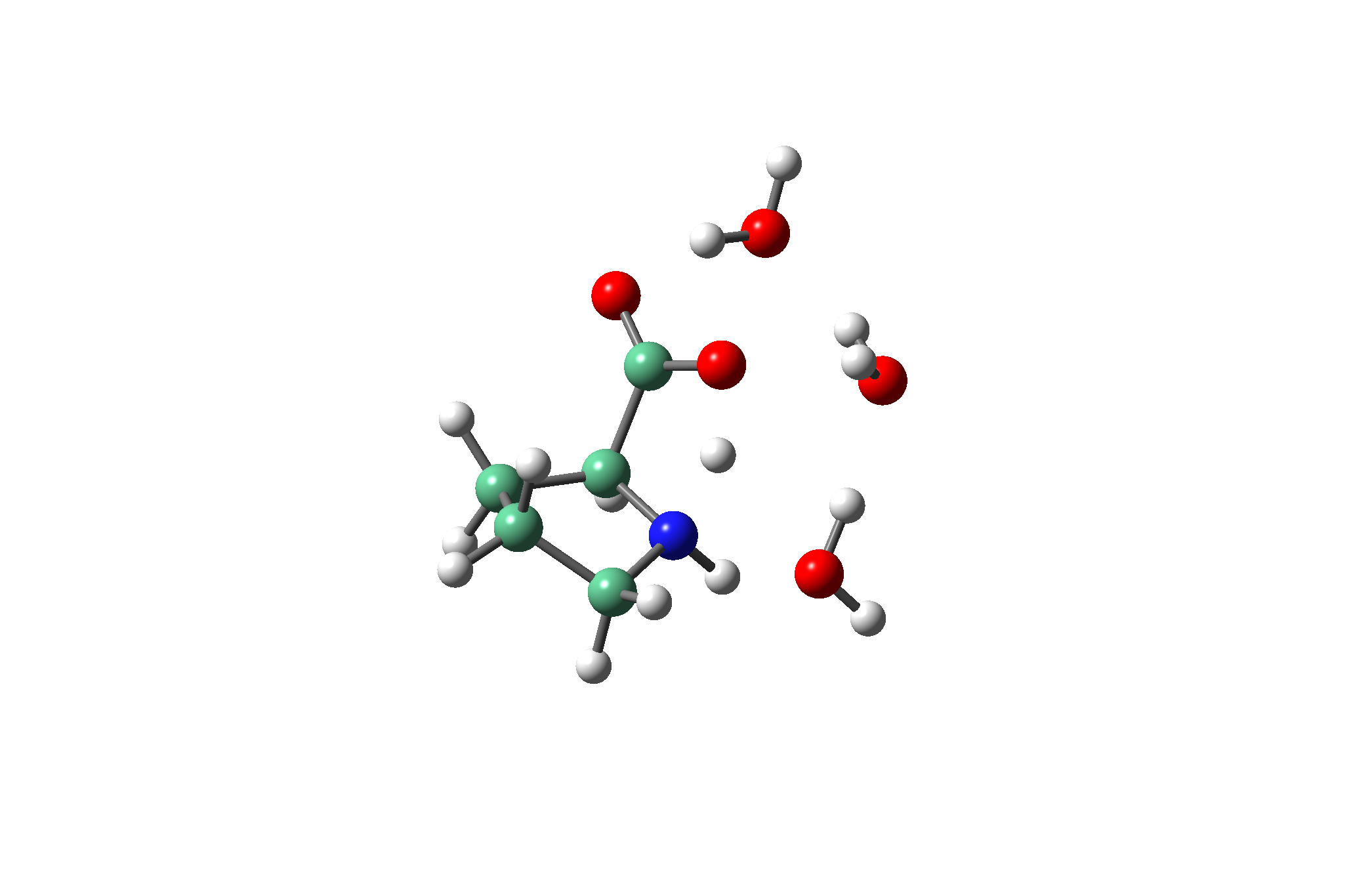

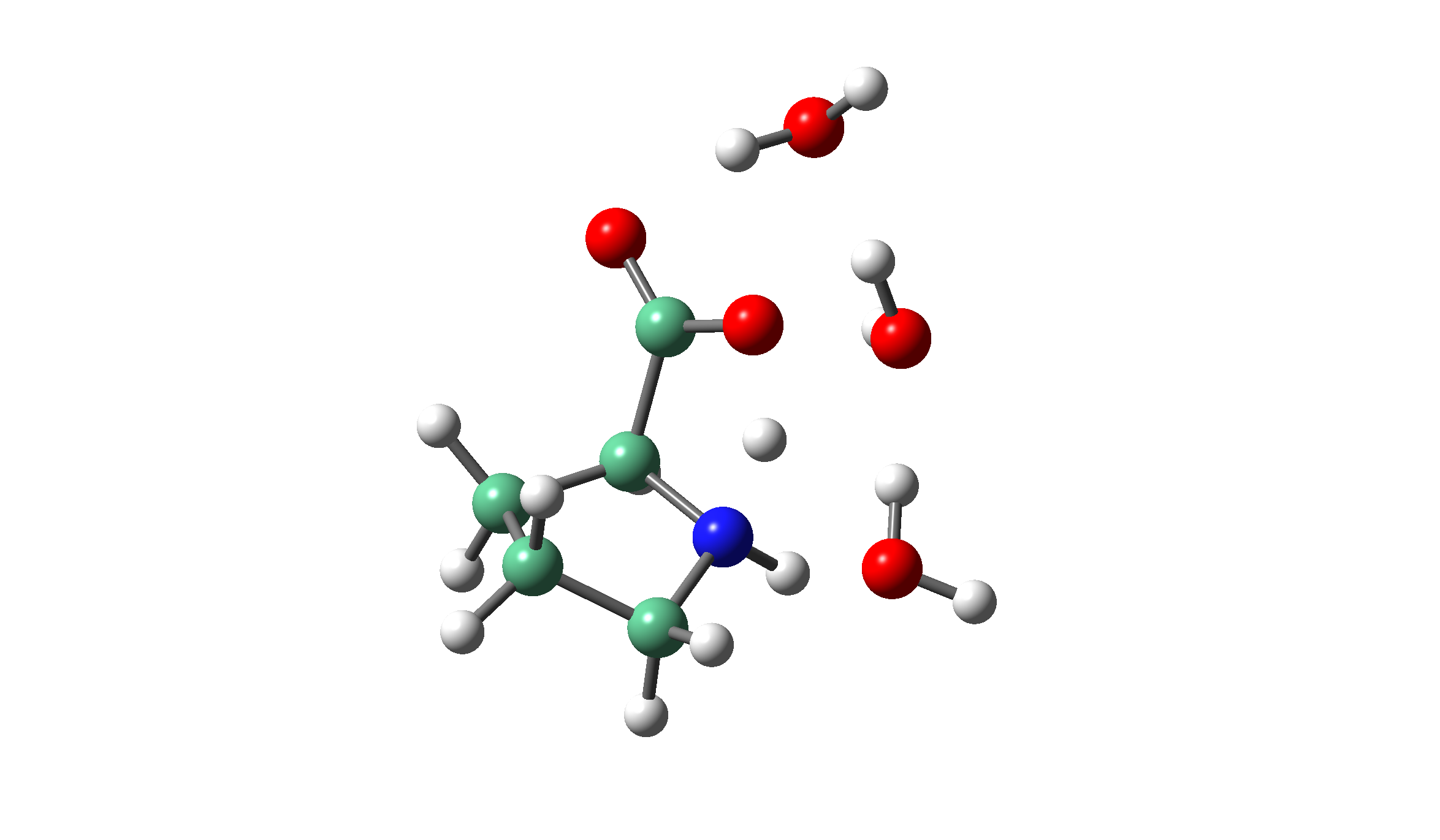

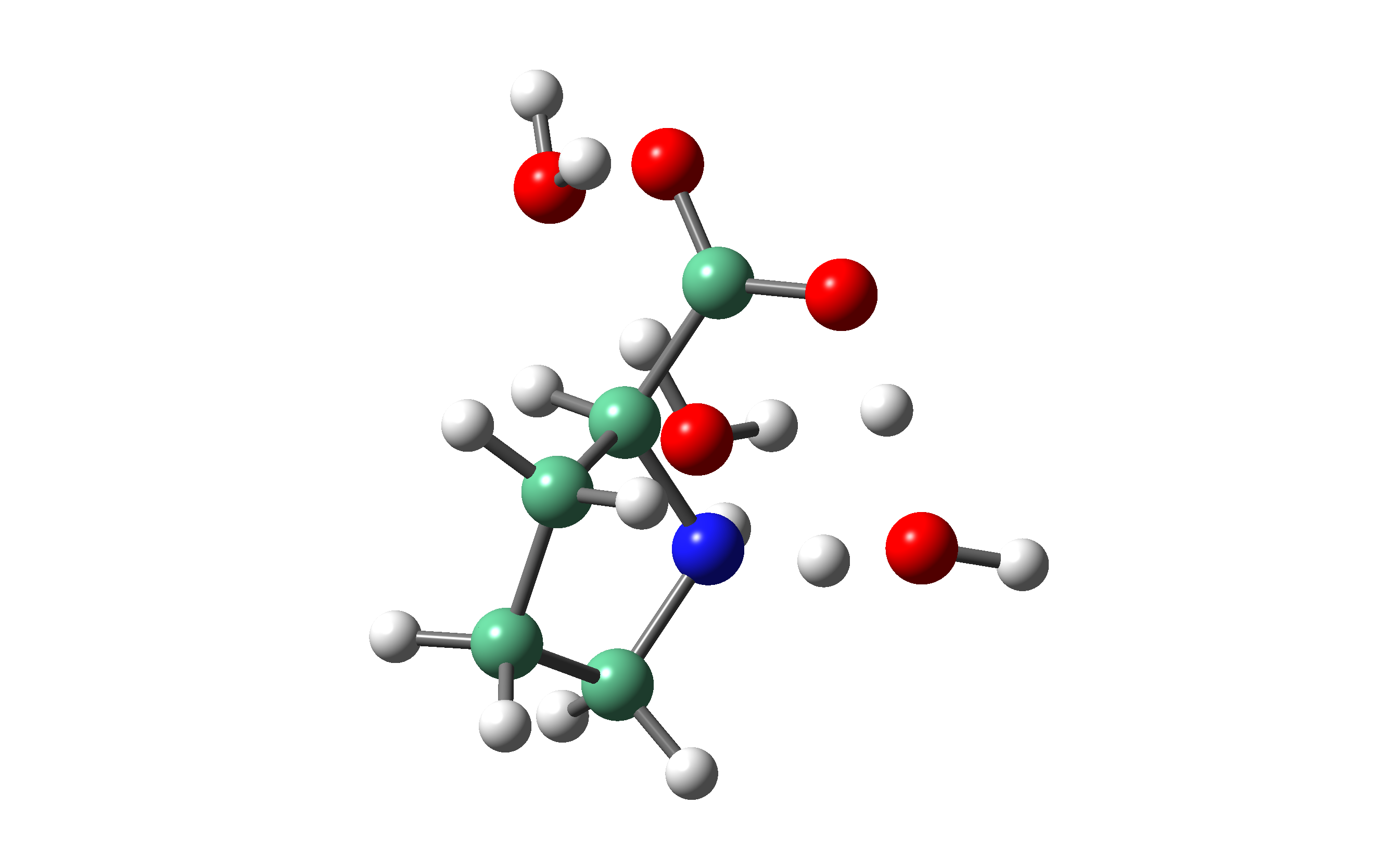

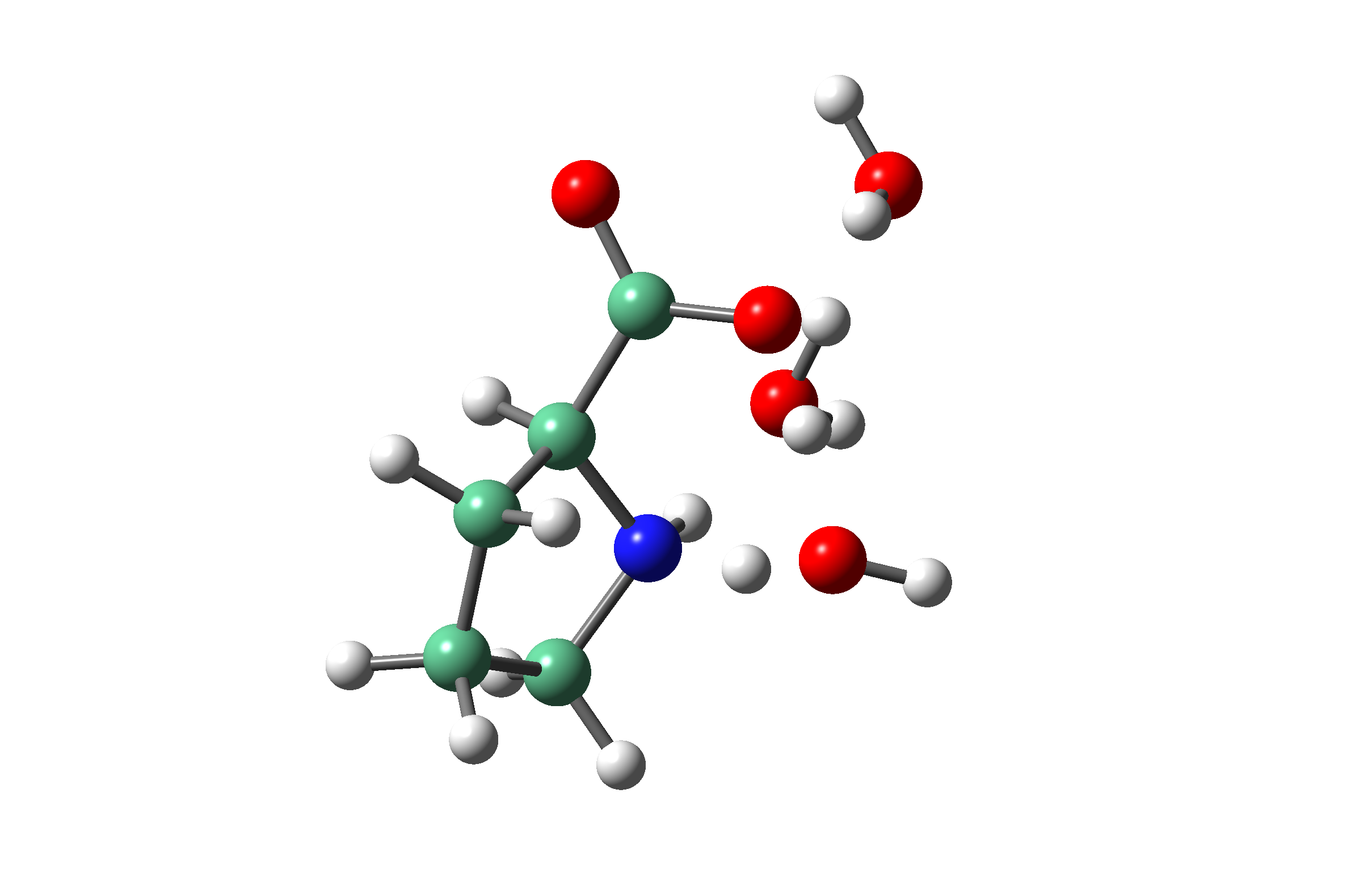


O1

O3

O4

O5

O2

N

O1

O3

O4

O5

O2

N

O2

O4

O5

O1

N

O1

O3

O5

O4

O2

N

O3

(a) **TS3W_II-I_** (3.8) (b) **TS3W_I-II_** (4.3)

H1

1.230

1.291

1.251

1.270

H1

1.133

1.298

1.227

1.271

1.142

1.233

1.265

1.286

(c) **TS3W_III-III_** (6.3) (d) **TS3W_III-IV_** (8.6)

(e) **TS3W_IV-V_** (2.6)


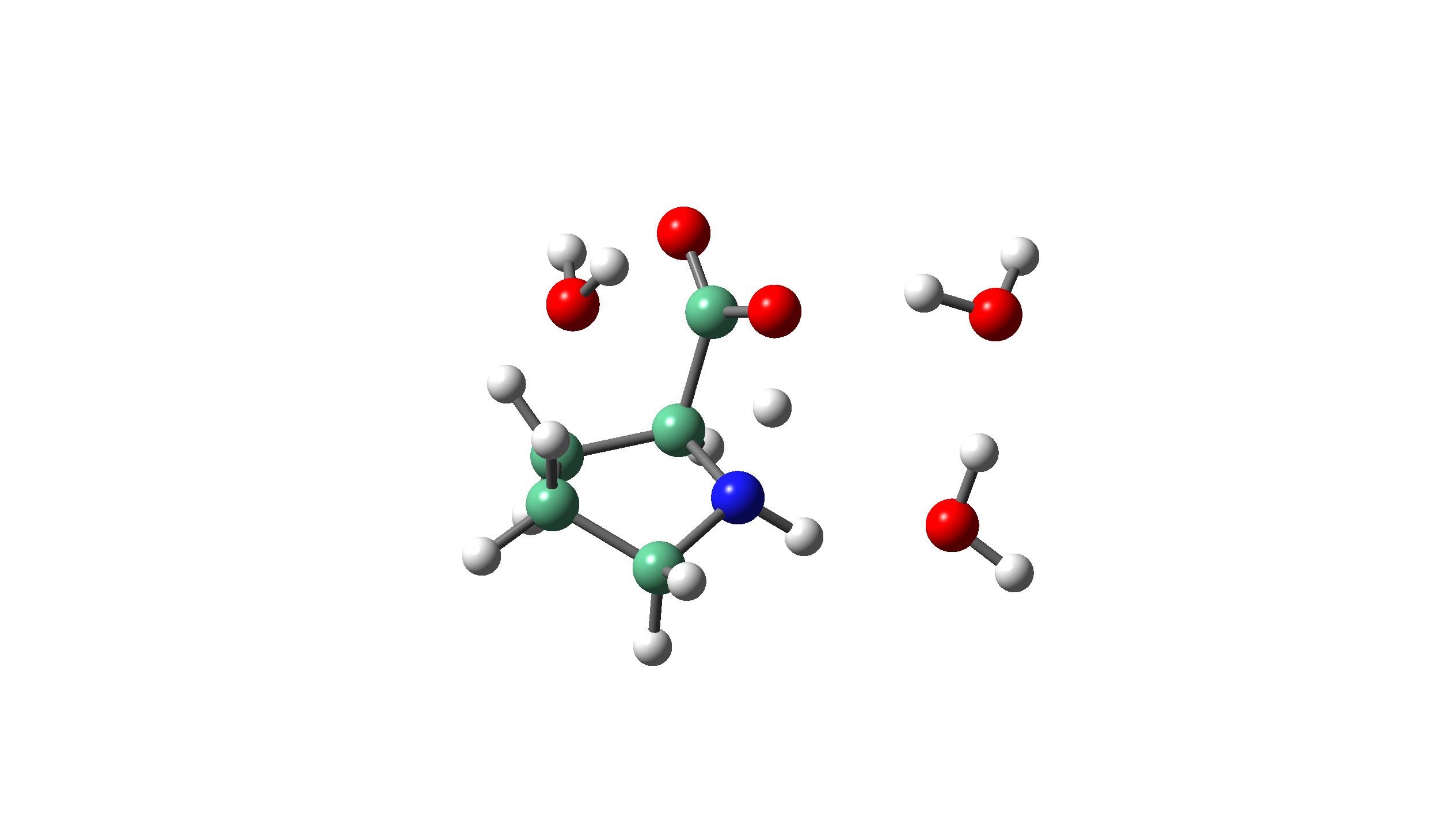


O1

O3

O4

O5

O2

N

1.203

1.316

**Figure S11.** Transition state structures for the conformational transformations from canonical to zwitterionic proline with presence of three water molecules. Activation barriers (kcal/mol) are given in parentheses. H-bonds (Å) are marked with dashed lines.


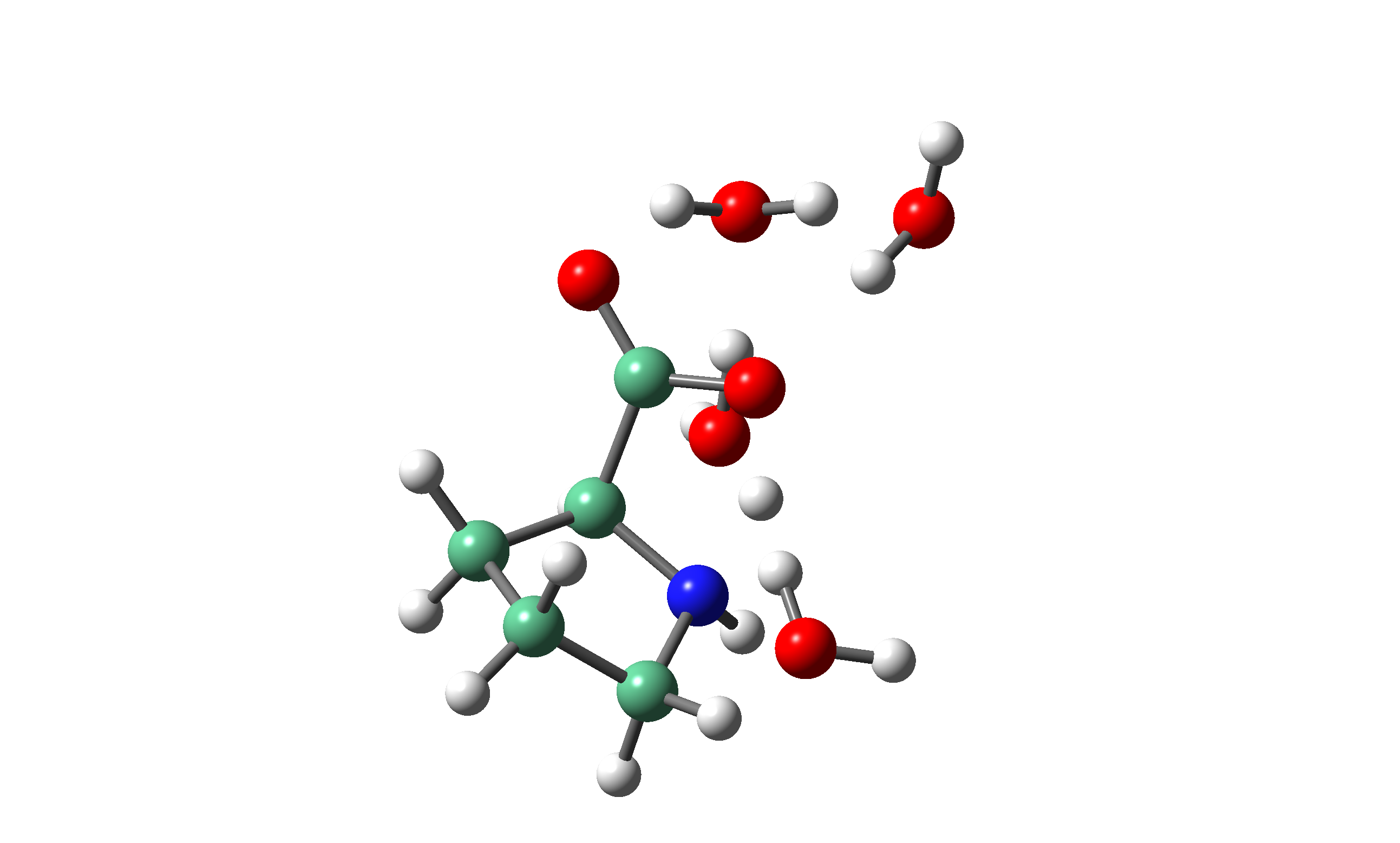

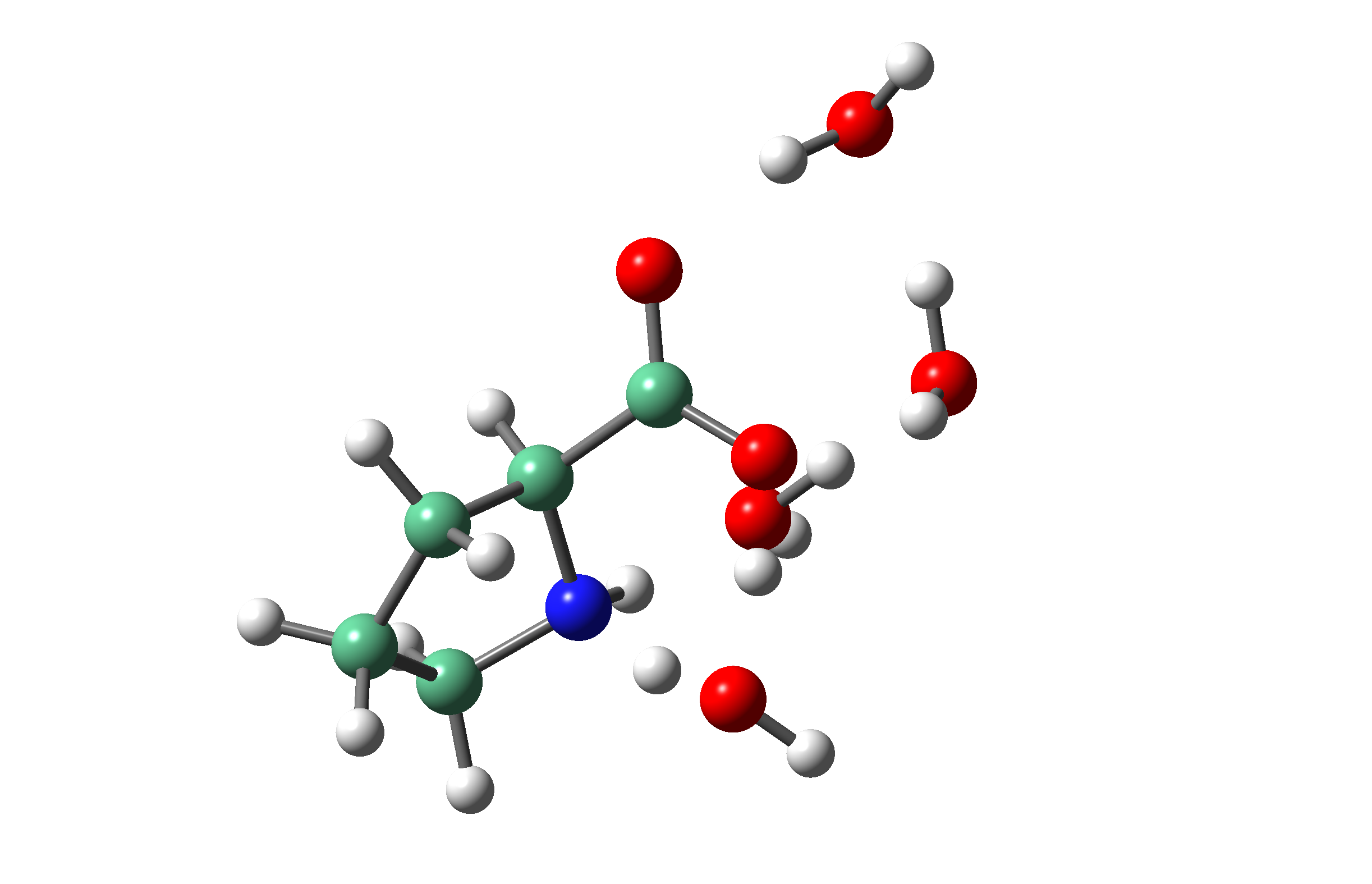

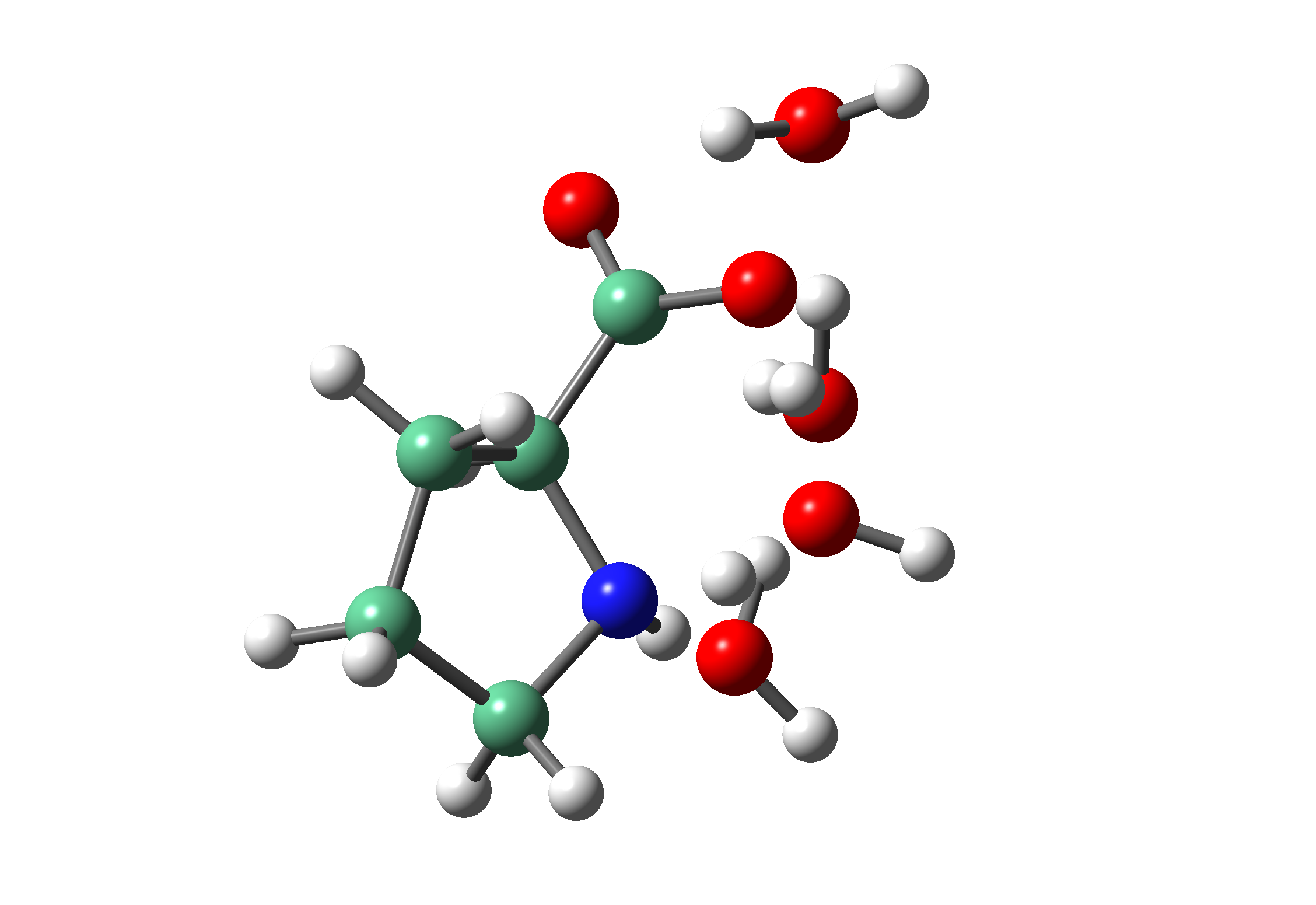

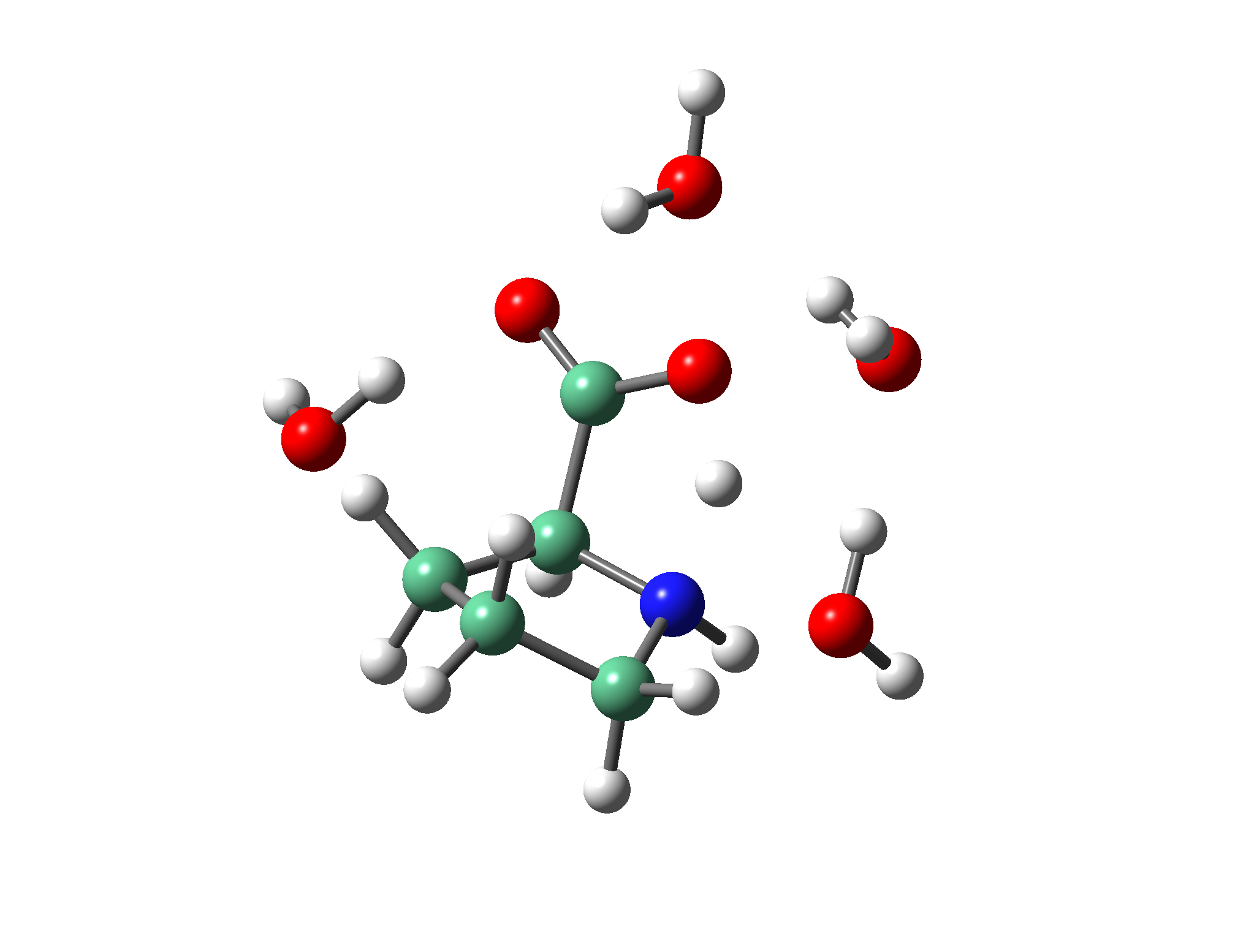


O1

O3

O6

O5

O2

N

O1

O4

O3

O5

O2

N

O1

O6

O4

O5

O2

N

O1

O3

O4

O6

O2

N

O3

(a) **TS4W_I-I_** (1.9) (b) **TS4W_II-I_** (5.8)

(c) **TS4W_II-III_** (5.3)  (d) **TS4W_III-IV_** (2.7)

O4

1.190

1.334

1.142

1.286

1.238

1.258

O6

1.131

1.300

1.258

1.238

1.188

O5

1.340

**Figure S12.** Transition state structures for the conformational transformations from canonical to zwitterionic proline with presence of four water molecules. Activation barriers (kcal/mol) are given in parentheses. H-bonds (Å) are marked with dashed lines.


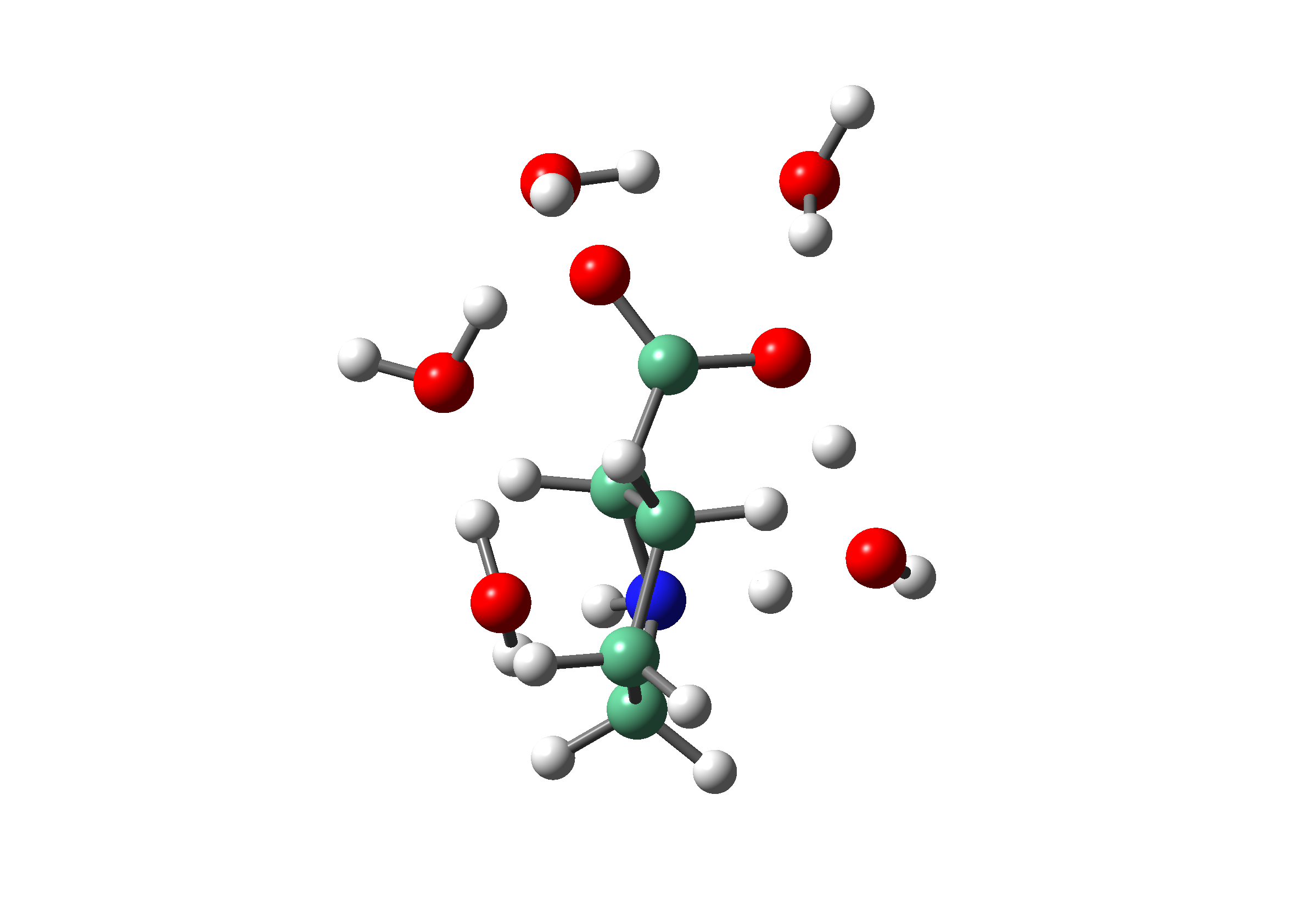

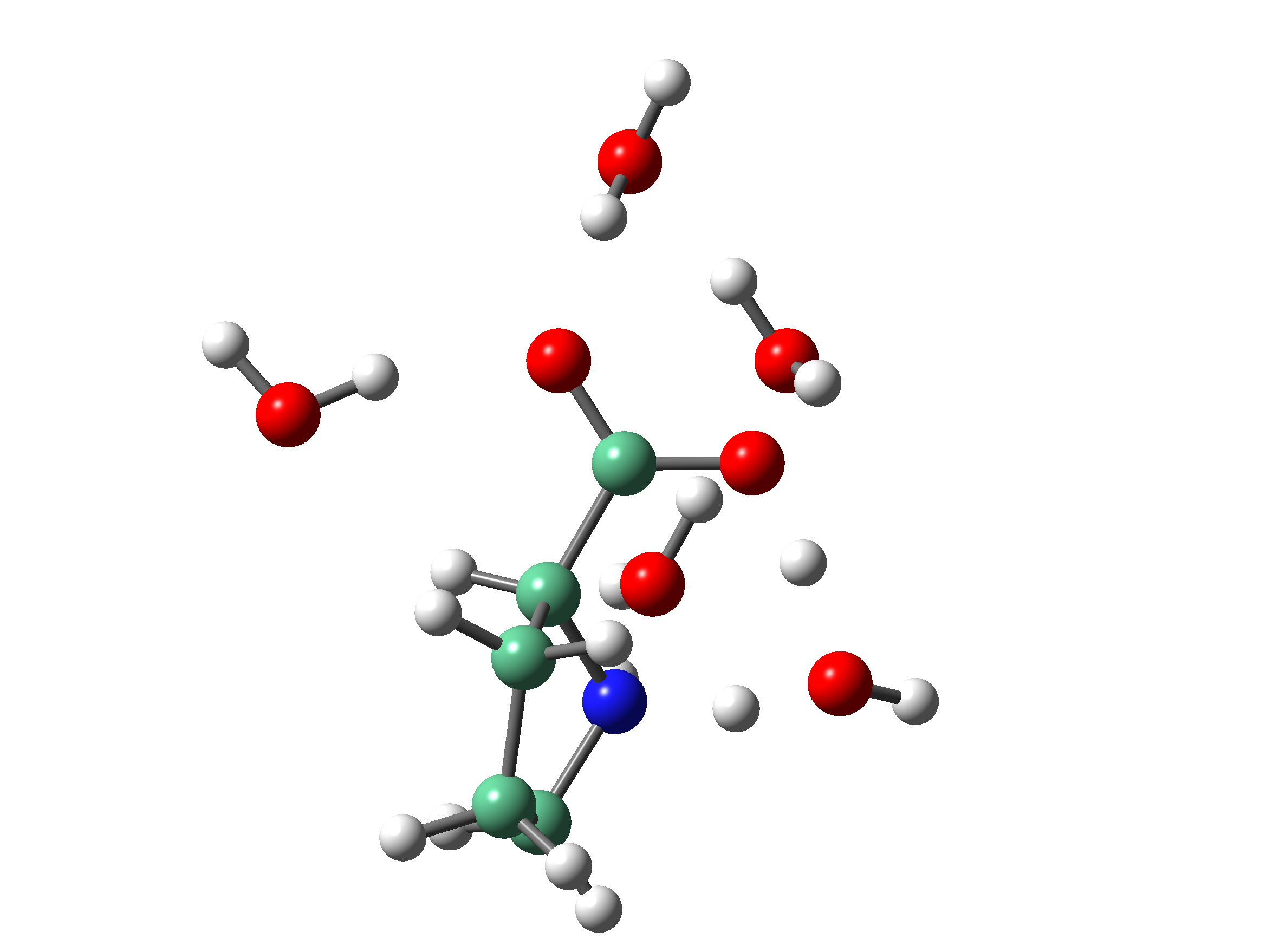


O1

O6

O6

O7

O2

O1

O3

O4

O5

O2

N

(a) **TS5W_I-I_** (4.2) (b) **TS5W_II-II_** (4.8)

O3

O4

O5

1.131

1.296

1.286

1.209

O7

1.142

1.284

1.298

1.201

**Figure S13.** Transition state structures for the conformational transformations from canonical to zwitterionic proline with presence of five water molecules. Activation barriers (kcal/mol) are given in parentheses. H-bonds (Å) are marked with dashed lines.
